# Bifidobacterial carbohydrate/nucleoside metabolism enhances oxidative phosphorylation in white adipose tissue to protect against diet-induced obesity

**DOI:** 10.1186/s40168-022-01374-0

**Published:** 2022-11-04

**Authors:** Gihyeon Kim, Youngmin Yoon, Jin Ho Park, Jae Won Park, Myung-guin Noh, Hyun Kim, Changho Park, Hyuktae Kwon, Jeong-hyeon Park, Yena Kim, Jinyoung Sohn, Shinyoung Park, Hyeonhui Kim, Sun-Kyoung Im, Yeongmin Kim, Ha Yung Chung, Myung Hee Nam, Jee Young Kwon, Il Yong Kim, Yong Jae Kim, Ji Hyeon Baek, Hak Su Kim, George M. Weinstock, Belong Cho, Charles Lee, Sungsoon Fang, Hansoo Park, Je Kyung Seong

**Affiliations:** 1 Department of Biomedical Science and Engineering, Gwangju Institute of Science and Technology (GIST), Gwangju, Korea; 2 Genome and Company, Pangyo-ro 255, Bundang-gu, Seongnam Korea; 3 Division of Nephrology, Department of Medicine, Chosun University Hospital, Chosun University School of Medicine, Gwangju, Korea; 4 Department of Family Medicine, Seoul National University Hospital, Seoul National University College of Medicine, Seoul, Korea; 5 Graduate school of Medical Science, Brain Korea 21 Project, Severance Biomedical Science Institute, Gangnam Severance Hospital, Yonsei University College of Medicine, Seoul, Korea; 6 Korea Basic Science Institute, Seoul Center, Seoul, South Korea; 7 The Jackson Laboratory for Genomic Medicine, Farmington, Connecticut 06032 USA; 8 Laboratory of Developmental Biology and Genomics, BK21 Plus Program for Advanced Veterinary Science and Research Institute for Veterinary Science, College of Veterinary Medicine, Seoul National University, Seoul, Korea; 9 Korea Mouse Phenotyping Center, Seoul National University, Seoul, Korea; 10 Department of Life Science, Ewha Womans University, Seoul, 03760 Korea; 11 The First Affiliated Hospital of Xi’an Jiaotong University, Xi’an, 710061 China; 12 Interdisciplinary Program for Bioinformatics, Seoul National University, Seoul, Korea

## Abstract

**Background:**

Comparisons of the gut microbiome of lean and obese humans have revealed that obesity is associated with the gut microbiome plus changes in numerous environmental factors, including high-fat diet (HFD). Here, we report that two species of *Bifidobacterium* are crucial to controlling metabolic parameters in the Korean population.

**Results:**

Based on gut microbial analysis from 99 Korean individuals, we observed the abundance of *Bifidobacterium longum* and *Bifidobacterium bifidum* was markedly reduced in individuals with increased visceral adipose tissue (VAT), body mass index (BMI), blood triglyceride (TG), and fatty liver. Bacterial transcriptomic analysis revealed that carbohydrate/nucleoside metabolic processes of *Bifidobacterium longum* and *Bifidobacterium bifidum* were associated with protecting against diet-induced obesity. Oral treatment of specific commercial *Bifidobacterium longum* and *Bifidobacterium bifidum* enhanced bile acid signaling contributing to potentiate oxidative phosphorylation (OXPHOS) in adipose tissues, leading to reduction of body weight gain and improvement in hepatic steatosis and glucose homeostasis. *Bifidobacterium longum* or *Bifidobacterium bifidum* manipulated intestinal sterol biosynthetic processes to protect against diet-induced obesity in germ-free mice.

**Conclusions:**

Our findings support the notion that treatment of carbohydrate/nucleoside metabolic processes-enriched *Bifidobacterium longum* and *Bifidobacterium bifidum* would be a novel therapeutic strategy for reprograming the host metabolic homeostasis to protect against metabolic syndromes, including diet-induced obesity.

Video Abstract

**Supplementary Information:**

The online version contains supplementary material available at 10.1186/s40168-022-01374-0.

## Introduction

Obesity is a multifactorial metabolic syndrome, and its prevalence is globally increasing [1, 2]. It has been widely reported that the gut microbiota could play pivotal roles in controlling host physiological homeostasis, including tissue inflammation, regulation of intermediary metabolism, and cellular energy metabolism [3, 4]. Especially, microbiota administration has been shown to change serum metabolites, such as short chain fatty acids (SCFAs) and branched-chain amino acids (BCAAs) to affect several diseases, including insulin resistance, inflammatory bowel disease (IBD), and colorectal cancer [5–7]. These interactions between host and the gut microbiota are associated with the host metabolism [8] and metabolic syndromes such as obesity, dyslipidemia, and hypertension are related to and lead chronic inflammation and alteration of gut microbial community [9–12].

The alteration of the gut microbiota has been widely accepted to control host metabolic pathways, such as glucose homeostasis, inflammation, and bile acid metabolism [13–15]. Reduced microbial richness of human individuals presents more pronounced dysregulated metabolic homeostasis [12, 16] and individualized variations in microbial composition correlates to the variability in glycemic responses [17]. Recent studies demonstrated that supplementation of specific gut microbiota, such as *Lactobacillus* spp. and *Bifidobacterium* spp. attenuates obesity and metabolic disorders through improving host metabolism [18–20].

Since microbiome interplay with human health, numerous efforts have been exploited to develop a specific strain of microbiome for therapeutic applications [21–24]. Indeed, we recently demonstrated that *Bifidobacterium bifidum*_K57, a specific strain of *Bifidobacterium bifidum*, is able to modulate host immune response to immunotherapy, implying that strain-dependent microbial molecular mechanisms are crucial to controlling host physiological homeostasis [25]. However, the detailed mechanisms of how strain-specific gut microbiome differently modulate host physiological homeostasis remain still unclear.

To address this question, here we aimed to identify detailed molecular mechanisms of strain-specific gut microbiota to reduce the diet-induced body weight gain. Using 99 Korean human stool samples, 16S rRNA-based analysis showed that the abundance of *Bifidobacterium longum* and *bifidum* is negatively associated with visceral adipose tissue (VAT), body mass index (BMI), waist circumstance (WC), γ-glutamyl transpeptidase (γGTP), blood triglyceride (TG), and fatty liver. Comprehensive genomic analysis showed that strain-specific carbohydrate and nucleoside metabolic processes are crucial to protect against diet-induced obesity. Administration of specific commercial *Bifidobacterium* strains, such as *Bifidobacterium longum*_MG723 and *Bifidobacterium bifidum*_MG731 reduced body weight gain, downregulated inflammation, lipid synthesis, glucose synthesis, and upregulated bile acid metabolism and energy metabolism in diet-induced obese mice. Finally, we identified that those two strains for anti-obesity distinctly modulate intestinal gene signatures in SPF and germ-free mice to protect against body weight gain. Our results clearly propose that screening of microbiota at the strain level for a therapeutic approach is required for further development of microbiome engineering to protect against metabolic syndromes, including diet-induced obesity.

## Results

### 16S rRNA sequencing and analysis of human stool samples predict obesity-related gut microbiome

**Fig. 1:**
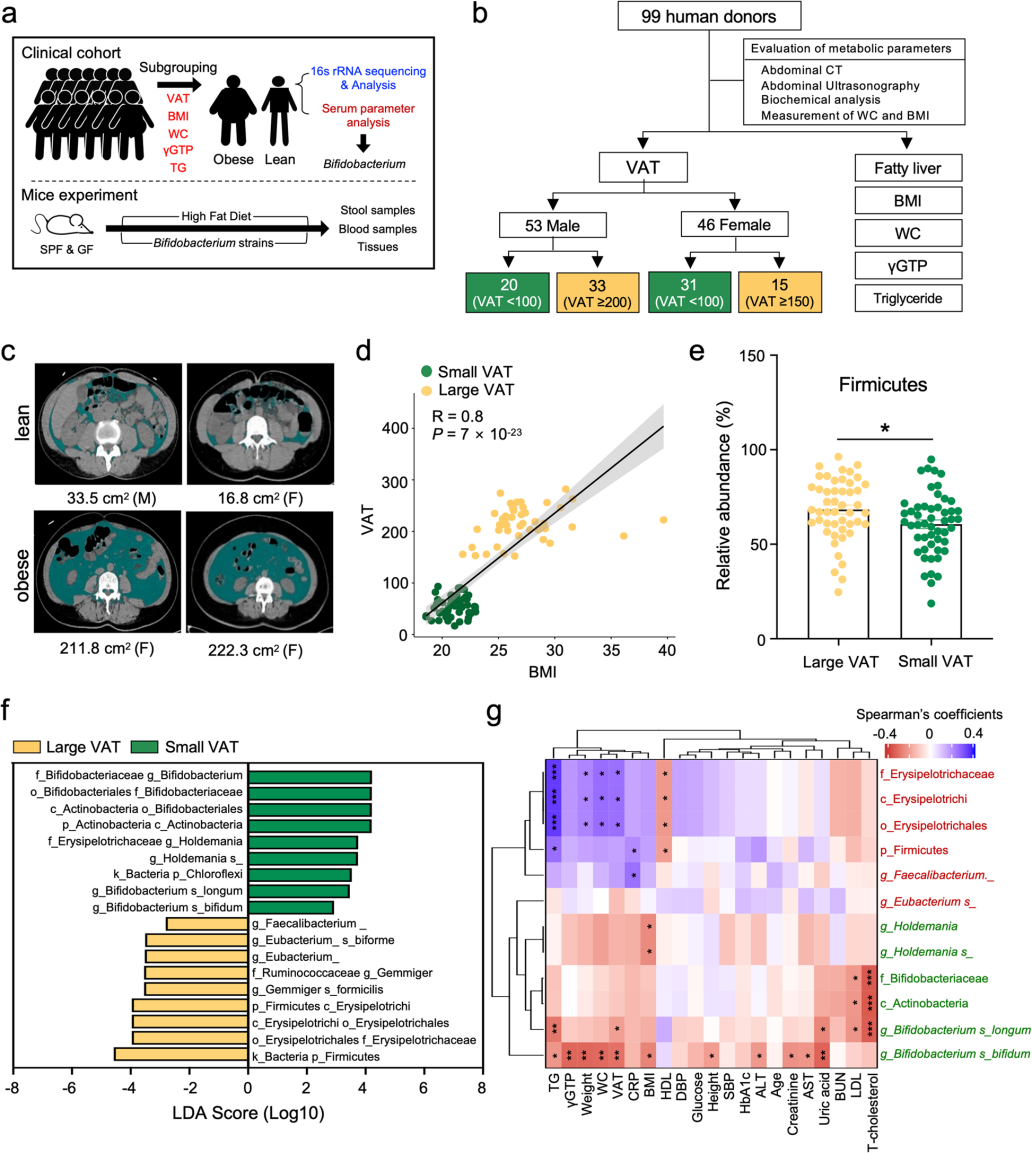
16S rRNA sequencing and analysis of human stool samples predict to find obesity-related gut microbiome. **a** Study design for human and animal experimental investigations. The clinical cohort was subgrouped by the visceral adipose tissue (VAT), body mass index (BMI), fatty liver, waist circumference (WC), γGTP, and blood triglyceride levels (TG). SPF, specific pathogen-free; GF, germ-free. Mice experiments were performed to investigate whether the *Bifidobacterium* strains protect against high-fat diet-induced obesity in mice. **b** Patient enrollment, treatment, and follow-up regimens. A total of 99 human samples were included in the analysis and were divided by various clinical indices. **c** Representative computed tomography (CT) measurement of VAT. M; male, F; female. **d** Pearson correlation between VAT and BMI in human donors. **e** Relative abundance of Firmicutes. Statistical analysis was performed using a two-sided unpaired *t* test. **f** A bar plot of linear discriminant analysis (LDA) scores from the linear discriminant analysis effect size (LEfSe) method illustrates the relative abundances of taxa that differ significantly between groups. **g** Heatmap of the Spearman’s rank correlation coefficient between 21 clinical markers and 12 obesity-associated taxa. green; high abundance in small VAT, red; high abundance in large VAT. CRP; C-reactive protein, T cholesterol; total cholesterol, HDL; high-density lipoprotein, LDL; low-density lipoprotein, SBP; systolic blood pressure, DBP; diastolic blood pressure, HbA1c; hemoglobin A1c, BUN; blood urea nitrogen, ALT; alanine aminotransferase. AST; aspartate aminotransferase, p; phylum, c; class, o; order, f; family, g; genus, s; species. For all graphs, *p<0.05

To examine microbiota composition in Korean population, we first performed 16S ribosomal RNA (rRNA) gene sequencing on stool samples collected from 99 donors (Fig. 1a) and obtained gut microbial genomic signature. Individuals were characterized by various metabolic parameters, including visceral adipose tissue (VAT), body mass index (BMI), fatty liver, waist circumference (WC), γGTP, and blood triglyceride levels (TG) (Fig. 1b). Central obesity, which is accumulated of the adipose tissue in the abdominal region (Fig. 1c), is associated with metabolic disorders, such as insulin resistance, cardiovascular diseases, and metabolic syndrome [26–29], and amounts of VAT were positively correlated with BMI in Korean individuals (Fig. 1d). We divided 99 human donors into obese and lean groups according to VAT to identify obesity-associated gut microbiome in the Korean population. The bioclinical characteristics and detailed clinical parameter information of each group have been provided (Supplementary Table 1). Though there were no changes in microbial gene counts and bacterial diversity between the large and small VAT groups in the Korean population (Supplementary Figure 1), we observed that the phylum Firmicutes was significantly enriched in the group with large VAT (Fig. 1e) as previously reported [30, 31]. Linear discriminant analysis (LDA) effect size indicated that the phylum Firmicutes and the class Erysipelotrichi were significantly enriched in the group with large VAT, whereas class Actinobacteria and genus *Holdemania* were significantly enriched in the group with small VAT (Fig. 1f). Notably, the *B. longum* and *B. bifidum* were markedly enriched in the small VAT group at the species level (Fig. 1f). Furthermore, the correlation analysis between metabolic parameters and gut microbiota exhibited that the abundance of *B. longum* and *B. bifidum* exhibited a negative correlation with metabolic markers, such as total cholesterol, alanine aminotransferase (ALT), aspartate aminotransferase (AST), triglyceride, γGTP, weight, waist circumference, and BMI (Fig. 1g). In addition, Actinobacteria showed a negative correlation with total cholesterol and low-density lipoprotein (LDL), and *Holdemania* also showed a negative correlation with BMI at the correlation analysis (Fig. 1g).

Even though most of the alpha diversity and beta diversity were not significantly different by the obesity-associated bioclinical characteristics except BMI for beta diversity, we observed consistent results with correlation analysis (Supplementary Figure 2, 3, 4 and 5). Both *B. longum* and *B. bifidum* were also more abundant in metabolically normal individuals based on BMI and fatty liver status (Supplementary Figure 2 and 5). *B. longum* was abundant in individuals with low TG levels whereas *B. bifidum* was abundant in individuals with low γGTP levels (Supplementary Figure 4), where a sole high-TG level participant had a high abundance of *B. longum*. Taken together, these data suggest that *B. longum* and *B. bifidum* are important microbial species negatively associated with obesity-related metabolic indices among Korean individuals.

### *B. longum* and *B. bifidum* protect against diet-induced obesity in a strain-dependent manner

**Fig. 2:**
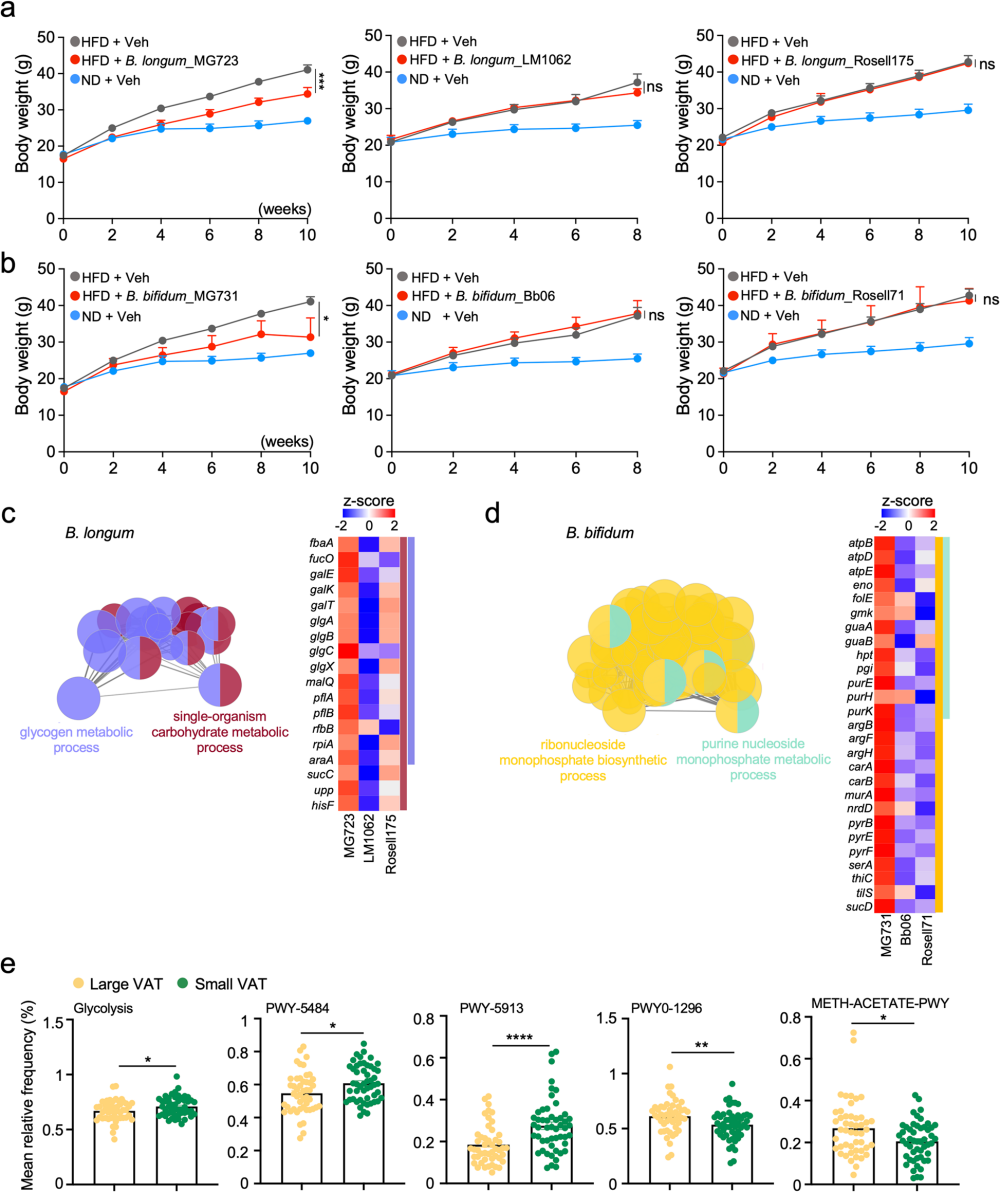
Strain-specific carbohydrate/nucleoside metabolic processes of *Bifidobacterium longum* and *Bifidobacterium bifidum* are crucial to protect against diet-induced obesity. **a** Bodyweight curves in normal diet (ND) and HFD-fed mice treated with vehicle and three different strains of *Bifidobacterium longum_*MG723, *Bifidobacterium longum_*LM1062, and *Bifidobacterium longum_*Rosell175 (*n* = 5 per group). Statistical analysis was performed using two-way ANOVA with Tukey’s multiple comparison. Data expressed as mean ± S.E.M. **b** Bodyweight curves in normal diet (ND) and HFD-fed mice treated with vehicle and three different strains of *Bifidobacterium bifidum_*MG731, *Bifidobacterium bifidum_*Bb06, and *Bifidobacterium longum_*Rosell71 (*n* = 5 per group). Statistical analysis was performed using two-way ANOVA with Tukey’s multiple comparison. Data expressed as mean ± S.E.M. Veh, vehicle is PBS. **c** Differentially regulated genes and pathways in the different strains of *Bifidobacterium longum.* Left panel: network representation of enriched Gene Ontology (GO) biological processes. Functionally related groups partially overlap. Right panel: heatmap of genes related to the pathway. **d** Differentially regulated genes and pathways in the different strains of *Bifidobacterium. bifidum.* Left panel: network representation of enriched Gene Ontology (GO) biological processes. Functionally related groups partially overlap. Right panel: heatmap of genes related to the pathway. *z* score was calculated to indicate the gene expression level. The statistical significance of network analysis for **c** and **d** was calculated using two-sided hypergeometric tests, and the false discovery rate was corrected using the Bonferroni step-down method. **e** A comparison of functional metagenome profiling was performed using the MetaCyc between small VAT (green, *n* = 51) and high VAT (yellow, *n* = 48) groups. The statistical analysis was performed using Wilcoxon-Mann-Whitney analysis. For all graphs, *p<0.05, **p<0.01, ***p<0.001, and ****p<0.0001. ns, non-significant

To determine whether *B. longum* and *B. bifidum* protect against HFD-induced obesity, we used three different strains of both *B. longum* (MG723, LM1062, and Rosell175) and *B. bifidum* (MG731, Bb06, and Rosell71), which were donated and commercially purchased. To examine the physiological roles of those microbiota to modulate host metabolic homeostasis, we administered each strain or PBS (as vehicle control) to mice fed with a high-fat diet (HFD). As expected, the control HFD-fed mice group exhibited a significant increase in body weight (Fig. 2a, b). While *B. longum*_LM1062 and Rosell175 were not able to decrease body weight gain in HFD-fed mice, only one strain *B. longum*_MG723 was shown to suppress diet-induced body weight gain (Fig. 2a). Likewise, only one strain *B. bifidum*_MG731 appeared to reduce body weight gain in HFD-fed mice whereas other two different *B. bifidum* strains, *B. bifidum*_Bb06 and Rosell71, were not able to reduce body weight gain (Fig. 2b). These data imply that strain-specific microbial molecular mechanisms are crucial to modulate host metabolic homeostasis to reduce body weight gain on HFD.

To investigate whether host physiological impacts of the microbiome to protect against HFD-induced obesity are strain-specific, we next performed transcriptomic analysis on all strains of the *B. longum* (MG723, LM1062, and Rosell175) and *B. bifidum* (MG731, Bb06, and Rosell71) in exponential phase*.* In three *B. longum* strains transcriptome, Gene Ontology (GO) of differentially expressed genes (DEGs; fold change > 2) showed that diverse metabolic processes related to glycogen metabolism and carbohydrate metabolism were significantly enriched in the strain of *B. longum*_MG723 compared to *B. longum*_LM1062 and Rosell175 (Fig. 2c and Supplementary Fig. 6). Next, we analyzed *B. bifidum* transcriptome from three different strains. We observed that the nucleoside monophosphate metabolic process was markedly enriched in the strain of *B. bifidum*_MG731 compared to *B. bifidum*_Bb06 and Rosell71 at the gene ontology of DEGs (Fig. 2d and Supplementary Fig. 6). Given that nucleoside monophosphate is a compound consisting of a nucleobase linked to either deoxyribose or ribose sugar which is a glycosyl donor for biosynthesis of carbohydrates [32], our bacterial transcriptome results suggest that microbial carbohydrate metabolic process is associated with reprogramming host metabolic homeostasis for the protection against diet-induced obesity on a strain-specific manner.

To explore the functional implications of the gut microbiome composition related to human obesity, we next compared the potential functional prediction using MetaCyc analysis of the 16S rRNA gene sequencing. We observed that pathways related to “glycolysis,” “glycolysis II (PWY-5484),” and “partial tricarboxylic acid (TCA) cycle (PWY-5913)” were enriched in the small VAT group, whereas the pathway of “purine ribonucleosides degradation (PWY0-1296)” and “methanogenesis (Meth-Acetate-PWY)” was enriched in the large VAT group (Fig. 2e). These findings are consistent with the idea that the microbial transcriptome could be relevant to 16S rRNA sequencing data from our human cohort, and microbial genes would be a predictable indicator for reprogramming host metabolic homeostasis to protect against diet-induced obesity.

### *B. longum*_MG723 and *B. bifidum*_MG731 promote OXPHOS in the adipose tissue by potentiation of bile acid signaling

**Fig. 3:**
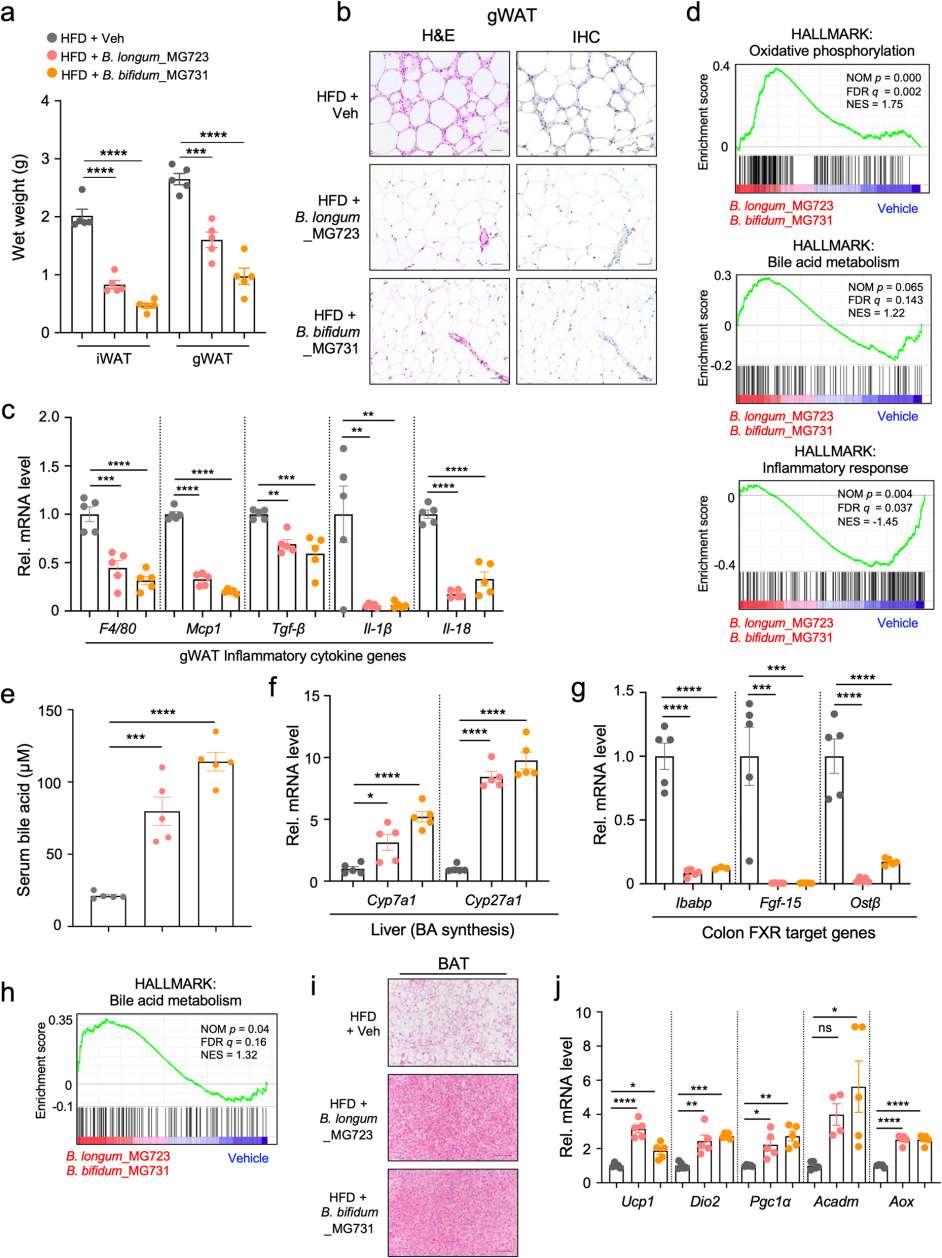
*B. longum_*MG723 and *B. bifidum_*MG731 enhance OXPHOS in white adipose tissues via potentiation of bile acid signaling. **a** Wet weights of inguinal WAT (iWAT) and gonadal WAT (gWAT) in HFD-fed mice treated with vehicle, *B. longum_*MG723 or *B. bifidum_*MG731 for 10 weeks (*n* = 5 per group). The statistical analysis was performed using one-way ANOVA with Tukey’s multiple comparison. Data expressed as mean ± S.E.M. **b** Histological analysis of gWAT. Left panel: hematoxylin and eosin (H&E) staining. Right panel: immunohistochemistry (IHC) images of macrophage. Scale bar, 100 μm. **c** Gene expression profile of inflammatory cytokines in gWAT (*n* = 5 per group). The statistical analysis was performed using one-way ANOVA with Tukey’s multiple comparison. Data expressed as mean ± S.E.M. **d** GSEA from gWAT transcriptome. **e** Serum bile acid pool size (*n* = 5 per group). The statistical analysis was performed using one-way ANOVA with Tukey’s multiple comparison. Data expressed as mean ± S.E.M. **f** Hepatic gene expression profile involved in bile acid synthesis (*n* = 5 per group). The statistical analysis was performed using one-way ANOVA with Tukey’s multiple comparison. Data expressed as mean ± S.E.M. **g** Colonic gene expression profile of FXR target genes (*n* = 5 per group). The statistical analysis was performed using one-way ANOVA with Tukey’s multiple comparison. Data expressed as mean ± S.E.M. **h** GSEA from the colonic transcriptome. **i** Representative brown adipose tissue (BAT) histological section images. Scale bar, 100 μm. **j** Gene expression profiles of thermogenesis and fatty acid oxidation in BAT (*n* = 5 per group). The statistical analysis was performed using one-way ANOVA with Tukey’s multiple comparison. Data expressed as mean ± S.E.M. For all graphs, *p<0.05, **p<0.01, ***p<0.001, and ****p<0.0001, ns, non-significant

Expansion of the adipose tissue by hyperplastic and/or hypertrophic growth has been shown to induce chronic inflammation and produce inflammatory cytokines that ultimately contribute to systemic metabolic dysregulation [33–35]. Oral treatment of *B. longum*_MG723 and *B. bifidum*_MG731 led to a decrease of wet weights of both inguinal white adipose tissue (iWAT) and gonadal adipose tissue (gWAT) in HFD-fed mice without any changes of food intake (Fig. 3a and Supplementary Fig. 7a). The cross-sectional area of adipocytes and crown-like structure (CLS) in the visceral fat tissue and infiltrated-macrophages were remarkably reduced in both *B. longum_*MG723 and *B. bifidum_*MG731-treated mice, implying that treatment of both strains markedly improved inflammation in WAT (Fig. 3b). Consistent with reduced adiposity and CLS, expression of the pro-inflammatory genes including *F4/80*, *Mcp1*, *Tgf-β*, *Il-1β*, and *Il-18* were largely reduced in visceral fat tissue from the *B. longum*_MG723 and *B. bifidum*_MG731-treated mice (Fig. 3c). Furthermore, reduction of macrophage population was also confirmed by transcriptomic profiling of immune cell population in WAT (Supplementary Fig. 7b). To address the functional changes of the visceral white fat tissue, we performed RNA sequencing analysis of the gonadal fat tissue. Gene Set Enrichment Analysis (GSEA) [36] revealed that gene sets involved in oxidative phosphorylation and bile acid metabolism were significantly enriched in the *B. longum_*MG723 and *B. bifidum_*MG731-treated mice (Fig. 3d and Supplementary Fig. 8a). These results suggested that treatment of *B. longum_*MG723 and *B. bifidum_*MG731 remodels physiological properties of the white adipose tissue from energy storage into energy expenditure by increased OXPHOS to protect against HFD-induced obesity. Consistent with histological analysis, GSEA revealed that inflammatory responses were remarkably reduced in the gWAT from *B. longum_*MG723 and *B. bifidum_*MG731-treated mice (Fig. 3d and Supplementary Fig. 8a).

Since bile acid metabolism was enriched in white adipose tissue by oral treatment of *B. longum_*MG723 and *B. bifidum_*MG731 (Fig. 3d), we next examined serum bile acid pool size. We observed that serum bile acid concentrations in *B. longum_*MG723 and *B. bifidum_*MG731-treated mice were significantly elevated compared with vehicle-treated mice (Fig. 3e). Consistently, expression of *Cyp7a1* and *Cyp27a1* genes that are involved in hepatic bile acid synthesis was markedly increased after oral treatment of *B. longum_*MG723 and *B. bifidum_*MG731 (Fig. 3f). It has been reported that fibroblast growth factor 15 (*Fgf-15*) represses hepatic bile acid synthesis [37–39]. Thus, we next evaluated the gene expression profiles of various FXR target genes, including *Fgf-15* in the intestinal tract. We observed that FXR target genes, *Ibabp*, *Fgf-15*, and *Ostβ*, were remarkably reduced in *B. longum_*MG723 and *B. bifidum_*MG731-treated mice (Fig. 3g). As a consequence, GSEA revealed that intestinal bile acid metabolism was also enriched in the intestinal tracts of mice treated with *B. longum_*MG723 and *B. bifidum_*MG731 (Fig. 3h and Supplementary Fig. 8b). These data clearly suggest that *B. longum_*MG723 as well as *B. bifidum_*MG731 reduced FXR-mediated *Fgf-15* expression in the intestine to increase hepatic bile acid synthesis.

Previously, the bile acid signaling pathway was reported to increase energy expenditure by promoting intracellular thyroid hormone activation in brown adipose tissue (BAT) [40–42]. The prominent accumulation of lipid vesicles was markedly reduced in both *B. longum_*MG723 and *B. bifidum_*MG731-treated mice (Fig. 3i). Gene expression analysis confirmed the induction of thermogenic genes *Ucp1*, *Dio2*, and *Pgc-1α*, as well as fatty acid oxidation genes *Acadm*, *Aox* in BAT from mice treated with *B. longum_*MG723 and *B. bifidum_*MG731 (Fig. 3j), whereas those of mice treated with *B. longum*_Rosell 175 were not significantly induced (Supplementary Fig. 9a). Altogether, our data clearly demonstrated that both *B. longum_*MG723 and *B. bifidum_*MG731 promoted basal thermogenic function of BAT to increase energy expenditure to improve metabolic homeostasis.

### *B. longum*_MG723 or *B. bifidum*_MG731 improves hepatic steatosis and glucose homeostasis in diet-induced obese mice

**Fig. 4:**
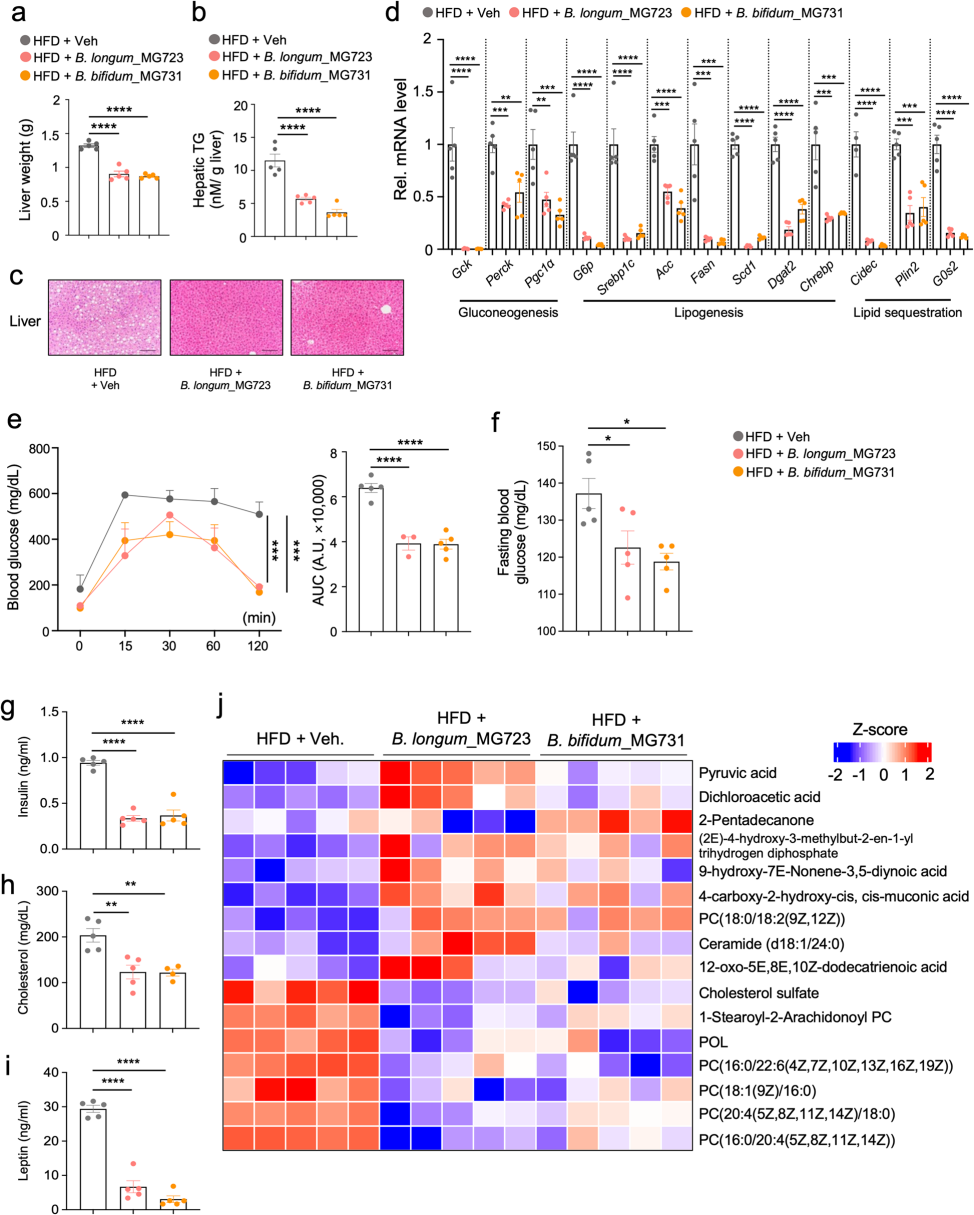
*B. longum_*MG723 and *B. bifidum_*MG731 improve hepatic steatosis and metabolic markers in HFD-induced obesity. **a** Wet weights of the liver in HFD-fed mice treated with vehicle, *B. longum_*MG723 or *B. bifidum_*MG731 for 10 weeks (*n* = 5 per group). The statistical analysis was performed using one-way ANOVA with Tukey’s multiple comparison. Data expressed as mean ± S.E.M. **b** Levels of hepatic triglycerides (TG) (*n* = 5 per group). The statistical analysis was performed using one-way ANOVA with Tukey’s multiple comparison. Data expressed as mean ± S.E.M. **c** Representative liver histological section images. Scale bar, 100 μm. **d** Hepatic gene expression profile (*n* = 5 per group). The statistical analysis was performed using one-way ANOVA with Tukey’s multiple comparison. Data expressed as mean ± S.E.M. **e** Glucose tolerance test (*n* = 5 per group). The statistical analysis was performed using two-way ANOVA with Tukey’s multiple comparison. Data expressed as mean ± S.E.M. **f** Fasting glucose level (*n*=5 per group). **g** Fasting serum insulin level (*n* = 5 per group). **h** Serum cholesterol level (*n* = 5 per group). **i** Serum leptin level (*n* = 5 per group). The statistical analysis was performed using one-way ANOVA with Tukey’s multiple comparison (**f–i**). Data expressed as mean ± S.E.M. **j** Serum metabolite concentrations measured by metabolomics analysis (*n* = 5 per group); POL, 1-palmitoyl-2-oleoyl-3-linoleoyl-rac-glycerol. For all graphs, *p<0.05, **p<0.01, ***p<0.001, and ****p<0.0001

Consistent with the reduction of body weight gain, oral treatment of *B. longum_*MG723 and *B. bifidum_*MG731 dramatically reduced liver wet weights in HFD-fed mice (Fig. 4a). Our human cohort data exhibited that abundance of *B. longum* and *B. bifidum* was correlated with fatty liver and metabolic parameters including AST and ALT (Fig. 1g and Supplementary Fig. 5). Similarly, hepatic triglyceride (TG) and hepatic fat deposition were remarkably decreased in *B. longum_*MG723 and *B. bifidum_*MG731-treated group (Fig. 4b and c). Hepatic gene expression profiling revealed that expression of gluconeogenic and lipogenic genes was decreased by oral treatment of *B. longum_*MG723 and *B. bifidum_*MG731 (Fig. 4d), but not by oral treatment of *B. longum*_Rosell175 (Supplementary Figure 9b). Glucose tolerance test revealed that reduction of hepatic steatosis and gluconeogenesis led to improved glucose homeostasis in mice treated with *B. longum_*MG723 and *B. bifidum_*MG731. (Fig. 4e, f). Consistent with improved glucose homeostasis, *B. longum_*MG723 and *B. bifidum_*MG731 treatment significantly improved endocrine and metabolic profiles, including blood insulin, cholesterol, and leptin (Fig. 4g–i), whereas *B. longum*_Rosell175 treatment did not exhibit any changes in those parameters (Supplementary Fig. 9c), indicating that oral treatment of *B. longum_*MG723 and *B. bifidum_*MG731 improved hepatic steatosis as well as glucose homeostasis and metabolic parameters.

Next, we examined *B. longum_*MG723 and *B. bifidum_*MG731-mediated changes of serum metabolites. Serum metabolite profiling showed that pyruvic acid and dichloroacetic acid were largely increased in *B. longum_*MG723-treated mice, and 2-pentadecanone was increased in *B. bifidum_*MG731-treated mice (Fig. 4j). Given that pyruvate and 2-pentadecanone are derived from glucose [43, 44], these results indicated that oral treatment of *B. longum_*MG723 and *B. bifidum_*MG731 promoted glucose metabolism by microbial carbohydrate metabolic process in a direct and an indirect manner. In addition, other metabolites derived from phosphatidylcholine, known to increase in obese organisms [45, 46], were largely reduced in both *B. longum_*MG723 and *B. bifidum_*MG731-treated mice compared to the vehicle-treated group (Fig. 4j). All these data clearly proposed that administration *of B. longum*_MG723 and *B. bifidum*_MG731 led to reduced levels of fasting glucose, insulin, cholesterol, and leptin with reduced hepatic gene expression involved in gluconeogenesis and lipogenesis in diet-induced obese mice.

### Colonization of *B. longum*_MG723 or *B. bifidum*_MG731 contributes to the reduction of body weight gain in HFD-fed germ-free mice

**Fig. 5:**
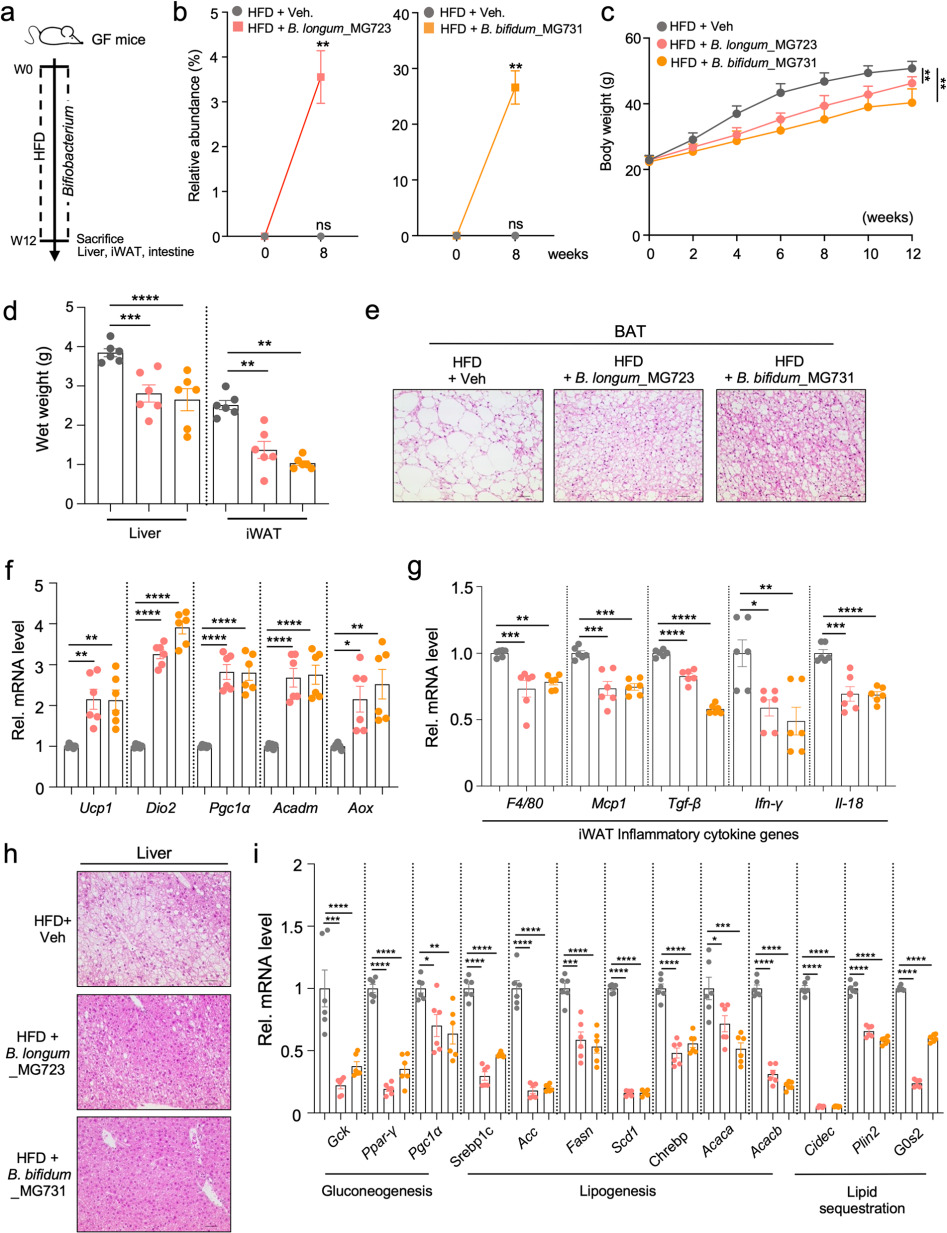
*B. longum_*MG723 and *B. bifidum_*MG731 ameliorate HFD-induced obesity in germ-free mice model. **a** Study design for animal works using HFD-fed germ-free mice treated with *B. longum_*MG723 and *B. bifidum_*MG731. **b** Relative abundance of *B. longum* and *B. bifidum* in HFD-fed germ-free mice (*n* = 6 per group). The statistical analysis was performed using two-way ANOVA with Tukey’s multiple comparison. Data expressed as mean ± S.E.M. **c** Body weight curves of HFD-fed germ-free mice treated with vehicle, *B. longum_*MG723 or *B. bifidum_*MG731 (*n* = 6 per group)*.* The statistical analysis was performed using two-way ANOVA with Tukey’s multiple comparison. Data expressed as mean ± S.E.M. **d** Wet weights of iWAT and liver (*n* = 6 per group). The statistical analysis was performed using one-way ANOVA with Tukey’s multiple comparison. Data expressed as mean ± S.E.M. **e** Representative brown adipose tissue (BAT) histological section images. Scale bar, 100 μm. **f** Gene expression profiles of thermogenesis and β-oxidation in BAT (*n* = 6 per group). The statistical analysis was performed using one-way ANOVA with Tukey’s multiple comparison. Data expressed as mean ± S.E.M. **g** Gene expression profiles involved in inflammatory responses in inguinal adipose tissues (*n* = 6 per group). The statistical analysis was performed using one-way ANOVA with Tukey’s multiple comparison. Data expressed as mean ± S.E.M. **h** Representative liver histological section images. Scale bar, 50 μm. **i** Hepatic gene expression profile involved in lipid and gluconeogenesis determined by qPCR (*n* = 6 per group). Data expressed as mean ± S.E.M. The statistical analysis was performed using one-way ANOVA with Tukey’s multiple comparison. For all graph, *p<0.05, **p<0.01, ***p<0.001, and ****p<0.0001

To address whether a single community of *B. longum_*MG723 or *B. bifidum_*MG731 is able to protect against diet-induced obesity without commensal gut microbiota, we orally administered *B. longum_*MG723 and *B. bifidum_*MG731 to HFD-fed germ-free mice for 12 weeks (Fig. 5a). We have observed that administration of *B. longum_*MG723 and *B. bifidum_*MG731 increased abundance of *B. longum* and *B. bifidum* in stool samples (Fig. 5b) and exhibited a distinct microbial community between groups (Supplementary Fig. 10), implying that both *B. longum_*MG723 and *B. bifidum_*MG731 stably colonized in the gut of germ-free mice. Consistent with our previous data from specific-pathogen-free (SPF) mice, *B. longum_*MG723 and *B. bifidum_*MG731 treatment exhibited a significant decrease in the body weight as well as wet weights of liver and fat tissue in HFD-fed germ-free mice (Fig. 5c, d). Histological analysis revealed that lipid droplet sizes were largely decreased in BAT from *B. longum_*MG723 and *B. bifidum_*MG731-treated germ-free mice (Fig. 5e). Similar to histological analysis, treatment of *B. longum_*MG723 and *B. bifidum_*MG731 increased expression of genes involved in thermogenesis and fatty acid oxidation in BAT (Fig. 5f). In the white adipose tissue, treatment of *B. longum_*MG723 and *B. bifidum_*MG731 reduced inflammatory cytokine gene expression in HFD-fed germ-free mice (Fig. 5g). Moreover, treatment of *B. longum_*MG723 and *B. bifidum_*MG731 improved hepatic steatosis (Fig. 5h) and repressed expression of genes involved in hepatic gluconeogenic and lipogenic genes (Fig. 5i) in HFD-fed germ-free mice. Taken together, both *B. longum_*MG723 and *B. bifidum_*MG731 increased basal energy expenditure in BAT and reduced WAT adiposity and hepatic steatosis to restore metabolic homeostasis in HFD-fed germ-free mice.

### Inhibition of cholesterol biosynthetic process of *B. longum*_MG723 or *B. bifidum*_MG731 is critical to protect against diet-induced obesity in germ-free mice

**Fig. 6:**
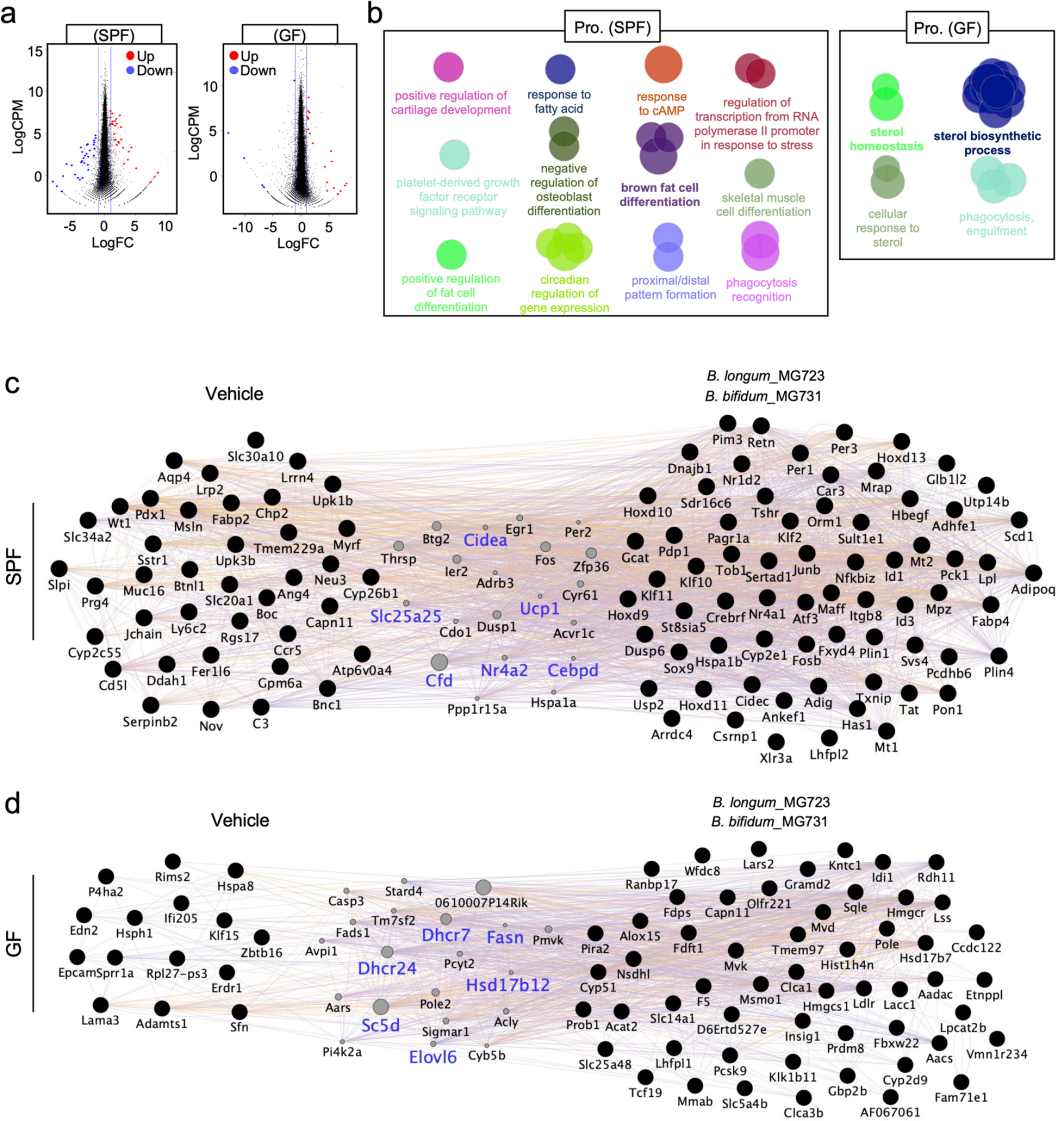
*B. longum_*MG723 and *B. bifidum_*MG731 attenuate intestinal cholesterol biosynthesis in the germ-free mice to protect against HFD-induced obesity. **a** Volcano plots of DEGs from HFD-fed SPF mice and germ-free mice treated with vehicle, *B. longum_*MG723 or *B. bifidum_*MG731 (*n* = 1–2 per group)*.* Genes for FDR < 0.05 and |log2(fold change)| > 1 were indicated in red or blue. **b** Network representation of enriched Gene Ontology (GO) biological processes, enriched in HFD-fed mice treated with *B. longum_*MG723 and *B. bifidum_*MG731 compared to HFD-fed mice treated with vehicle. The statistical significance was calculated using two-sided hypergeometric tests, and the false discovery rate was corrected using the Bonferroni step-down method. **c** Genetic network enriched in vehicle and probiotics (*B. longum*_MG723 and *B. bifidum*_MG731) from HFD-fed SPF mice. **d** Genetic network enriched in vehicle and probiotics (*B. longum*_MG723 and *B. bifidum*_MG731) from HFD-fed germ-free mice

To determine molecular mechanisms of how *B. longum_*MG723 and *B. bifidum_*MG731 modulate intestinal homeostasis to reprogram metabolic homeostasis, we next performed intestinal transcriptome analysis in SPF and germ-free mice treated with vehicle or *B. longum_*MG723 and *B. bifidum_*MG731. When comparing differentially expressed genes (DEGs), we observed that gene expression patterns were distinct between the probiotics-treated group and vehicle-treated group of both SPF and germ-free mice (Fig. 6a). Pathway analysis revealed that the gene signature involved in brown fat cell differentiation was enriched in HFD-fed SPF mice treated with *B. longum_*MG723 and *B. bifidum_*MG731 (Fig. 6b). Interestingly, we observed that sterol homeostasis and sterol biosynthetic process were enriched only in probiotics-treated HFD-fed germ-free mice (Fig. 6b), implying that both *B. longum_*MG723 and *B. bifidum_*MG731 differently modulate intestinal homeostasis in SPF and germ-free mice.

Finally, we analyzed intestinal molecular and genetic networks in DEGs from the probiotics-treated group and vehicle-treated group from both SPF and germ-free mice. In HFD-fed SPF mice, we have observed that distinct two DEGs between vehicle and *B. longum_*MG723- and *B. bifidum_*MG731-treated group were genetically connected by genes involved in BAT-mediated thermogenesis, such as *Ucp1*, *Cidea*, *Slc25a25*, and *Thrsp*, suggesting that treatment of *B. longum_*MG723 and *B. bifidum_*MG731 modulated intestinal genetic network to control BAT-mediated thermogenesis for reprogramming metabolic homeostasis (Fig. 6c). Interestingly, distinct two DEGs between vehicle and *B. longum_*MG723- and *B. bifidum_*MG731-treated group in germ-free mice were connected by genes involved in fatty acid and cholesterol biosynthesis, such as *Dhcr7*, *Dhcr24*, *Sc5d*, *Hcd17b12*, *Elovl6*, and *Fasn* (Fig. 6d). As consistent, GSEA showed that cholesterol homeostasis was upregulated in vehicle-treated group in germ-free mice (Supplementary Fig. 11). Taken together, our data clearly suggest that *B. longum_*MG723 and *B. bifidum_*MG731 reprogram metabolic homeostasis via BAT-mediated thermogenesis (in SPF mice) or fatty acid and cholesterol biosynthesis (in germ-free mice).

## Discussion

Previous studies have indicated that the gut microbiome can affect the obesity and obesity-related metabolic disorders by regulating the energy expenditure and absorption from the diet [47, 48]. The body mass index (BMI) is commonly used as a measure of classifying overweight and obesity in adults [49]. However, BMI has several limitations, including not reflecting the location and amount of body fat [49], and visceral fat accumulation is an independent risk factor for cardiovascular diseases [50, 51]. Therefore, we determined the VAT of 99 human donors using computed tomography (CT) and divided obese and lean groups according to VAT amounts. Microbial community analysis of individual stool samples demonstrated that the abundance of *B. longum* and *B. bifidum* are markedly enriched in individuals with a low amount of VAT, as well as individuals with low body mass index (BMI) and no fatty liver.

Primary bile acids are produced from cholesterol in the liver and released into the small intestine [52, 53]. Our results have shown that oral treatment of *B. longum*_MG723 and *B. bifidum*_MG731 markedly increased the expression of Cyp7a1 and Cyp27a1 genes that are involved in hepatic bile acid synthesis (Fig. 3f). These results demonstrated that *B. longum*_MG723 and *B. bifidum*_MG731 reduced diet-induced cholesterol level through increasing cholesterol metabolism. The gut microbiome not only modified primary bile into secondary bile acids, but bile acids also influence the gut microbial community [53, 54]. Previous studies demonstrated that bile acids influence host glucose and lipid metabolism in multiple tissues, such as the liver, brown, and white adipose tissues [52, 55]. Moreover, bile acids regulate the immune system by impacting T cell differentiation [56, 57]. Oral treatment of *B. longum*_MG723 and *B. bifidum*_MG731 inhibits inflammation in white adipose tissues (Figs. 3b–d and 5g) and promotes the expression of genes involved in thermogenesis in brown adipose tissues (Figs. 3i and 5f). Our results demonstrated that oral treatment of *B. longum*_MG723 and *B. bifidum*_MG731 changes the bile acid metabolism, which is highly associated with suppressing the diet-induced obesity. Through in vitro and in vivo experiments, we also suggested that specific strains of *B. longum* and *B. bifidum* enhanced OXPHOS in the white adipose tissue via enhancing bile acid signaling to protect against diet-induced metabolic syndromes, including obesity and hepatic steatosis (Supplementary Fig. 12). Further studies should investigate whether other changes, which caused by *Bifidobacterium*, may lead to those phenotypic evidence against metabolic syndromes.

We demonstrated that protection against diet-induced obesity by treatment of microbiota in HFD-fed mice was largely dependent on a strain-specific manner. Bacterial transcriptome analysis showed carbohydrate metabolic process was enriched in a *B. longum* strain (*B. longum*_MG723) compared with other *B. longum* strains (*B. longum*_LM1062 and *B. longum*_Rosell175). We noticed that UDP-galactose-4-epimerase (*Gale*) was markedly enriched in the strain *B. longum*_MG723 (Fig. 2c). Given that UDP-galactose-4-epimerase plays a pivotal role to control nucleotide-sugars and glycosylation [58], *B. longum*_MG723 would modulate diverse enzymatic reactions to reprogram host metabolic homeostasis. Likewise, we also noticed that *SerA* gene expression was remarkably upregulated in *B. bifidum*_MG731 (Fig. 2d), which catalyzes the nucleoside metabolic process (serine biosynthesis) [59]. Given that serine biosynthesis is tightly linked to the glycolytic pathway [60, 61], upregulated microbial nucleoside metabolic processes may contribute to enhanced carbohydrate metabolism in both *B. longum_*MG723 and *B. bifidum*_MG731. As consistent, the MetaCyc results from human 16S rRNA data showed that partial TCA cycle, glucose, and glucose-1 phosphate degradation pathway were enriched in lean individuals, whereas purine ribonucleoside degradation pathway was enriched in obese individuals. Therefore, our MetaCyc data and microbial transcriptome analysis demonstrated that gut microbiota of lean individuals ameliorated metabolic syndromes, including obesity by enhancing carbohydrate/nucleoside metabolic processes. Notably, we found that pathway of methanogenesis from acetate was enriched in obese individuals (Fig. 2e). Given that dietary carbohydrate effects on methane production and elevation of intestinal methane is associated with higher BMI in obese individuals [62], enriched microbial carbohydrate metabolic processes would modulate host intestinal genetic functions to reduce gut availability of carbohydrate for methane production in lean individuals.

l-arginine is a semi-essential amino acid and crucial for glucose metabolism [63]. Previous studies have demonstrated that arginine supplementation modulated glucose metabolism, leading to improved insulin resistance and diet-induced obesity [63–65]. Consistently, we noticed that the expression of microbial genes, such as *ArgB*, *ArgF*, and *ArgH* involved in the arginine biosynthetic pathway was specifically enriched in *B. bifidum*_MG731 compared with other *B. bifidum* strains (*B. bifidum*_Bb06 and *B. bifidum*_Rosell71) (Fig. 2d). Taken together, these results suggested that both *B. longum_*MG723 and *B. bifidum*_MG731 may promote amino acid metabolism as well as carbohydrate/nucleoside metabolic processes to suppress diet-induced body weight gain.

The gut microbiome plays an important role in the absorption of intestinal cholesterol by regulating the production of coprostanone and coprostanol [66], which are difficult to absorb molecules from cholesterol [67]. The production of coprostanone and coprostanol excretes cholesterol into feces and it leads to reduce the blood cholesterol levels [67]. Additionally, previous studies discovered that some bacterial strains, including *Bacteroides* sp. strain D8, exhibit cholesterol-lowering effects [68, 69]. Our intestinal transcriptome analysis of GF mice showed that cholesterol biosynthetic process and cholesterol homeostasis (Fig. 6b and d, Supplementary Fig. 11) were enriched in HFD-fed mice compared to *B. longum_*MG723- and *B. bifidum_*MG731-treated mice. These results suggested that oral treatment of *B. longum_*MG723 and *B. bifidum_*MG731 inhibited diet-induced obesity by suppression of regulation of cholesterol absorption and synthesis in the intestine. However, the detailed mechanisms of intestinal cholesterol regulation by oral treatment of *B. longum_*MG723- and *B. bifidum_*MG731 need to be further studied.

## Conclusions

In summary, we have shown that specific microbiome species were associated with the improvement of diet-induced obesity through screening of gut microbiota of the Korean fecal samples, and the treatment with commercial probiotics could lead to the development of potential therapeutic strategies for the treatment of diet-induced obesity. Moreover, we have also revealed that the difference in strain-specific gene signatures contributes to strain-specific host metabolic homeostasis. Altogether, screening of microbial strain-specific host physiological homeostasis represents an attractive therapeutic strategy and warrants further comprehensive evaluation for clinical application for the treatment of metabolic syndromes, including obesity and hepatic steatosis.

## Methods

### Human donor characteristics

Donors, who visited the health-screening program at the Seoul National University Hospital (SNUH) between February 2017 and June 2017 (*n* = 99), were enrolled in this study. They went through various medical check-ups, including physical examination, blood test, abdominal CT, abdominal ultrasonography, questionnaire, and stool collection. A total of 99 eligible donors confirmed no serious diseases at the time of examination, and stool samples were analyzed for microbiome composition. Based on the visceral adipose tissue (VAT), the obese group was defined as more than 200 cm^2^ in males (*n* = 33) and more than 150 cm^2^ in females (*n* = 15), and the lean group was defined as less than 100 cm^2^ (*n* = 51). VAT was measured at the level of lumbar vertebra 4 or 5 using abdominal CT. Blood, stool, and radiologic tests were agreed upon by the donors and conducted at the Seoul National University Hospital. Clinical characteristics were indicated in Supplementary Table 1.

### Fecal sample collection and DNA extraction

All fecal samples were kept at −80°C until DNA extraction. DNA was extracted from fecal samples using FastDNA® SPIN Kit for Soil (MP Biomedicals, Solon, CA, USA) according to manufacturer’s recommendations. NanoDrop One Spectrophotometer (Thermo Scientific, DE, USA) was used for estimating DNA purity and quantity.

### Bacterial 16S rRNA sequencing of fecal samples

Bacterial 16S rRNA V3-V4 region was amplified by PCR according to the Illumina 16S rRNA Sequencing Library Preparation guide (Illumina, CA, USA), using the primers with adapter overhang sequences added [70].

Forward primer:

5′-TCGTCGGCAGCGTCAGATGTGTATAAGAGACAGCCTACGGGNGGCWGCAG-3′ Reverse primer:

5′-GTCTCGTGGGCTCGGAGATGTGTATAAGAGACAGGACTACHVGGGTATCTAATCC-3′

Twenty-five microliters of PCR contained 2 μl of genomic DNA, 0.5 μl of each primer, 12.5 μl of 2X KAPA HiFi HotStart ReadyMix (Kapa Biosystems, MA, USA), and 9.5 μl of distilled water. The PCR conditions were as followed: 95 °C for 3 min, 25 cycles to denature the DNA, 50 °C for 30 s to annealing, 72°C for 30 s to extension, and final extension at 72°C for 5 min. The PCR products were cleaned up using AMPure XP Beads (Beckman Coulter, CA, USA). Dual indices’ attachment and Illumina sequencing adapters were performed by amplicon PCR product DNA (5 μl), Illumina Nextera XT Index Primer 1 (5 μl, N7xx), Nextera XT Index Primer 2 (5 μl, S5xx), 2X KAPA HiFi HotStart Ready Mix (25 μl), and nuclease-free water (10 μl) with thermocycling at 95°C for 3 min, 8 cycles of 95°C for 30 s, 55°C for 30 s, 72 °C for 30 s, and final extension at 72 °C for 5 min. PCR products were cleaned up using AMPure XP beads, and quality control of performed 16S rRNA libraries was performed using the Agilent Technologies 2100 Bioanalyzer (Agilent, CA, USA). Libraries were standardized and pooled for sequencing on the MiSeq platform (Illumina, CA, USA) according to the standard Illumina sequencing protocol.

### 16S rRNA sequencing and analysis

The quality of the raw sequence reads was analyzed using FastQC [71]. Illumina adapter sequences of the paired-end reads were removed using Cutadapt version 2.2 [72]. Then, the trimmed sequences were processed using QIIME2 version 2020. 8. Briefly, the reads were assigned to each sample according to a unique index; pairs of reads from the original DNA fragments were merged using an import tool in QIIME2 [73]. Quality control and trimming were performed to yield sequences with lengths of 270 and 210 bp for the forward and reverse reads, respectively. To remove low-quality bases at the end of the reads, the DADA2 software package [74] wrapped in QIIME2 was applied. To remove chimeras from the FASTQ files, a consensus method implemented in DADA2 was used. Alpha and beta diversity were analyzed using core-metrics-phylogenetic in the QIIME2 diversity plugin. Alpha and beta diversities were calculated using alpha- and beta-group significance in the QIIME2 diversity plugin, respectively. Alpha diversity was calculated by Shannon Index, and beta diversity was compared by principal coordinate analysis using Bray–Curtis distances. The significance of similarity among the groups was evaluated using permutational multivariate analysis of variance (PERMANOVA) with 999 permutations. Taxonomic annotation was performed by mapping the training reference set with primers (forward, 5′-CCTACGGGNGGCWGCAG-3′; reverse, 5′-GACTACHVGGGTATCTAATCC-3′) and extracting the V3–V4 region using GreenGenes version 13_8 [75]. Linear discriminant effect size analysis (LEfSe) was performed to identify differential features at the species level between groups based on linear discriminant analysis (LDA) scores using Galaxy implementation [76]. Correlation plots and calculations were generated in R studio with the ggplot2 package [77]. Linear discriminant effect size analysis (LEfSe) [76] compares abundances of bacterial species levels between obese and lean groups with a linear discriminant analysis (LDA) score. The MetaCyc pathways from 16S rRNA community were obtained by PICRUSt2 plug-in Qiime2 (v2019. 10.) with default setting [78]. The frequency of MetaCyc pathways was calculated using the Statistical Analysis of Metagenomic Profiles (STAMP) software [79].

### Mice experiments

All experiments described in this study were approved by the Institutional Animal Care and Use Committee (IACUC) of the CHA University and Seoul National University and performed in accordance with the guidelines presented by IACUC. All mice were housed in a temperature-controlled facility under a 12-h light-dark cycle. For high-fat diet (HFD)-fed specific-pathogen-free mice, male C57BL/6N mice (6 weeks old) were obtained from Orient Bio (Gapyeong, Gyeonggi, Korea). The mice were fed a normal diet (ND) (2018, Envigo, Indianapolis) or a high-fat diet (HFD) (60% calories from fat, D12492, Research diets, New Brunswick). For bacterial administration, mice were orally treated with PBS or bacteria every day for 8–10 weeks. Body weights were measured once a week. For HFD-fed germ-free mice model, male C57BL/6N mice were housed in germ-free isolators at the Korea Mouse Phenotyping Center, Seoul National University. The 8-week-old GF mice were fed HFD (60% calories from Fat, Research Diet, Cat# D18110605i) with PBS or bacteria for 12 weeks. Body weights were measured once a week. Stool samples were collected once a week. All animal experiments have been replicated in at least two more biological replicates (*n* = 5/group). All dots in the figures related to the mouse experiment indicate each mouse.

### Bacterial preparation and administration

*Bifidobacterium longum* MG723 (*B. longum_*MG723) and *Bifidobacterium bifidum* MG731 (*B. bifidum_*MG731) were donated from Mediogen (South Korea). *Bifidobacterium longum_*LM1062 (*B. longum_*LM1062) was donated from Lactomason (South Korea). *Bifidobacterium longum*_Rosell175 (*B. longum_*Rosell175) and *Bifidobacterium bifidum* Rosell71 (*B. bifidum_* Rosell71) were purchased from Lallemand (Canada). Each bacteria species was resuspended in PBS, and 1× 10^9^ CFU of bacteria was orally administered once a day for 8–12 weeks.

### Quantitative real-time RT-PCR analysis

The total RNA was extracted from frozen tissues using TRIzol reagent according to the manufacturer’s instructions. The purity and concentration of the total RNA were determined by NanoDrop one spectrophotometer (Thermo Fisher). One microgram of the total RNA was reverse transcribed using PrimeScript™ 1st strand cDNA synthesis kit (TAKARA, Japan). A real-time PCR was performed using CFX384 Touch real-time PCR System (BioRad) with SYBR Premix Ex Taq (TAKARA, Japan) and gene-specific primers. The comparative threshold cycle (CT) was used to analyze the relative changes in gene expression normalized against 36B4 mRNA expression. Primer sequences used in this study are listed in Supplementary Table 2.

### Serum glucose measurement

For glucose tolerance tests, 2 g of glucose per kilogram of mice body weight was injected intraperitoneally to overnight fasted mice. Serum glucose was measured using Accu-Check Performa Glucometer (Roche) by tail bleeds, and blood glucose was measured at intervals of 15 min for 2 h. The fasting glucose was measured at 6 weeks from the start of the mice experiment.

### Serum insulin assay

Serum insulin was assessed in a 96-well microplate using a commercial Morinaga Ultra-Sensitive Mouse/Rat Insulin ELISA Kit. (MIoBS, Japan) and measured A450 with a microplate reader (Molecular device, California, USA).

### Serum leptin assay

Serum leptin was assessed in a 96-well microplate using a commercial Morinaga Mouse/Rat Leptin ELISA Kit. (MIoBS, Japan) and monitored A450 with a microplate reader.

### Serum total cholesterol and triglyceride analysis

Serum concentrations of total cholesterol and triglyceride were analyzed by automated apparatus (Mindray BS-200, Shenzhen, China).

### Total bile acid (TBA) measurement

The bile acid determination in serum that was collected after centrifugation of the mice blood was performed with a spectrophotometric enzymatic assay. The assay was conducted according to the kit instructions (MAK382, Sigma-Aldrich, Inc, St Louis, Mo).

### Liver tissue triglyceride measurement

Liver extracts were prepared and incubated in a buffer containing 5%NP-40/ddH2O solution, and supernatants containing the triglycerides were separated. Triglyceride concentration was determined on the supernatant fraction using a commercial colorimetric assay kit (ab65336, Abcam, Paris, France).

### H&E and immunohistochemistry staining

All tissues were fixed in 4% paraformaldehyde overnight at 4°C and then the tissues were sequentially dehydrated in ethanol with increasing concentrations ranging from 50 to 100%. Dehydrated tissues were infiltrated with 100% xylene and embedded into paraffin. Then, the tissues were sectioned into thick slices (4 μm) and stained with hematoxylin and eosin (H&E). For immunohistochemistry, paraffin-embedded gonadal fat tissue sections were de-paraffinized and re-hydrated with 0.05% Triton X-100 in phosphate-buffered saline (PBS), and non-specific binding sites were blocked using Peroxidase Blocking Reagent (DAKO®, USA). Sections were then incubated with 1:100 diluted primary antibodies, including anti-F4/80 (ab111101, Abcam), overnight at 4 °C. Biotinylated secondary antibodies and peroxidase/DAB, rabbit/mouse were applied according to the manufacturer’s instructions (ChemMate™ DAKO EnVision™ Detection Kit, DakoCytomation). Color development was induced by incubation for 3 min with the 3,3′-diaminobenzidine (DAB) substrate. Specific staining was visualized by light microscopy. Adipocyte size was measured by ZEN software (Blue edition, Zeiss, Germany) in H&E-stained adipose tissue from gWAT. The assessment of the degree of fatty liver was evaluated according to the scoring system for rodents [80]. All histologic slides were evaluated twice by a pathologist (M. N.) blinded to the experimental details.

### Transcriptome analysis from mouse tissues

RNA from mice gonadal fat and intestine tissue were extracted using RNeasy Mini Kit (QIAGEN). Illumina HiSeq 2500 instruments were used for whole transcriptome sequencing (WTS). RNA-seq reads from tissue WTS were aligned to the mouse reference genome (GRCm38) using the STAR aligner [81]. Gene expression levels were quantified by RSEM [82]. Count values of RSEM were normalized and calculated using edgeR to obtain DEGs between HFD- and probiotic-treated mice (FDR < 0.05 for volcano plot, *p* value < 0.01 for biological pathway analysis, and gene network analysis). The DEGs were used for performing ClueGO to find the biological pathway. ClueGO is a tool, which is plugged-in CytoScape, combining GO terms to create functionally grouped annotations in a network [83]. GO Biological Process database of *Mus musculus* was used for functional enrichment analysis. Significantly enriched GO terms were calculated by a two-sided hypergeometric test with a Bonferroni correction (*P* < 0.05), and the degree of connectivity between terms in the network was calculated and were grouped based on a kappa score greater than 0.4 with a network specificity of 4–10. TPM (transcripts per kilobase million) were calculated through RSEM following STAR alignments. TPM values were used to identify gene sets enriched in samples showing obesity inhibition effect or with obesity and GSEA was performed using java GSEA desktop application (GSEA v2.1.0) [36]. Gene sets that showed upregulated or downregulated with an FDR < 0.25 were considered significant. To investigate the fraction of infiltrating immune cells in the mouse tissue, CIBERSORT (Cell type Identification By Estimating Relative Subsets Of known RNA Transcripts) (https://cibersort.stanford.edu/) analysis was conducted with default setting [84].

### Metabolomic analysis of mouse serum

Isopropanol was added to 50 μL of serum. After shaking for 3 h at 4 °C, the mixtures were centrifuged at 13,000×g for 5 min at 4 °C. The supernatant was diluted with an equal volume of deionized water and injected into an ultra-performance liquid chromatography/quadrupole time-of-flight mass spectrometry (UPLCQ/TOF–MS, Synapt G2Si, Waters, USA) system. UPLC separation was performed using an Acquity UPLC CSH C18 column (2.1 mm × 100 mm, 1.7 μm, Waters). Mobile phase A consisted of 10-mM ammonium formate and 0.1% formic acid in acetonitrile to water (6:4), whilst mobile phase B contained 10-mM ammonium formate and 0.1% formic acid in isopropanol to acetonitrile (9:1). The samples were eluted using the following conditions: initial 40% B to 53% at 2 min, to 50% A at 2.1 min, to 54% B at 12 min, to 70% B at 12.1 min, to 1% B at 18 min, 40% B at 18.1 min, and equilibrated for an additional 2 min. The flow rate was 0.4 ml/min. The column temperature was maintained at 55 °C. The mass acquisition was performed in both positive (ESI+) and negative (ESI−) electrospray ionization modes with the following parameters: capillary voltage of 2.0 kV, cone voltage of 10 V, source temperature of 120 °C, desolvation temperature of 550 °C, and desolvation gas flow of 900 L/h. Mass data were collected in the range of m/z 60–1400 with a scan time of 0.25 s and an inter-scan time of 0.02 s for 12 min. Mass data, including retention time, m/z, and ion intensities, were extracted using Progenesis QI software packages (Waters). The aligned and normalized data sets were analyzed by multivariate statistical analysis of SIMCA-P ver. 12.0+ (Umetrics, Umea, Sweden). Partial least-square discriminant analysis (PLS-DA) was used to visualize discrimination among groups fed ND, HFD, HFD + *B. bifidum* MG731, and HFD + *B. longum* MG731. The reliability correlation [p (corr)] values of all metabolites from the S-plot of the OPLS-DA between ND and HFD were extracted using the first component. Metabolites satisfying the following criteria were selected as potential markers: (a) cutoff values for the covariance of *p* ≥ │0.02│and for the correlation of *p* (corr) ≥ 0.5 in the OPLS-DA discrimination between ND and HFD and (b) fold change of 1.5 or more between ND and HFD. Metabolites were searched by the human metabolomics database (http://www.hmdb.ca/) and METLIN (http://metlin.scripps.edu/) based on mass spectra.

### Bacterial transcriptome sequencing

We performed transcriptomic sequencing of the bacterial strains in their exponential phase. Bacterial strains were cultured in agar medium at 37 °C for 48 h under anaerobic conditions. After colony isolation from the agar plate, the colony was cultured in broth medium at 37 °C for 48 h under anaerobic conditions. Subsequently, the broth medium was centrifuged and the supernatant was removed. Bacterial RNA was extracted using the ZymoBIOMICS RNA Miniprep Kit (Zymo Research. Sequencing and library construction were performed on the Illumina Hiseq 2500 with 101-bp paired-end. Ribosomal RNA was removed using the Ribo-Zero™ rRNA Removal Kit (Bacteria) (Epicentre). Libraries were prepared with the TruSeq RNA Sample Prep kit v2 (Illumina). RNA-sequenced reads were mapped on the reference genome of *Bifidobacterium longum* (NCTC_11818) and *bifidium* (NCTC_13001) using STAR with alignIntron MAX 1[81]. Then, the mapped reads were used to calculate read counts of genes using cufflinks [85], and the gene list was input into Cytoscape plug-in ClueGO v2.5.4 [83] to annotate functionally grouped networks. Functionally related GO terms for biological processes, cellular components, and molecular function in *Escherichia coli* (version: 18 November 2016) were grouped based on a kappa score greater than 0.4 with a network specificity of 4–10. The statistical significance was calculated using two-sided hypergeometric tests, and the false discovery rate was corrected using the Bonferroni step-down method.

### Statistical analysis

Statistical calculations were performed using Prism 9.3.1 (GraphPad), and statistical methods were provided in the figure legends. The statistical analysis of different groups is performed using Wilcoxon-Mann-Whitney test, Student’s *t* test, and ANOVA with Tukey’s multiple comparison test. Differences were considered to be statistically significant at values of *p* < 0.05. Details were indicated in each figure.

## Supplementary Information

**Additional file 1: Supplementary Figure 1**. Comparison of alpha and beta diversity of stool samples in human donors using visceral adipose tissue (VAT). a,b, Alpha (a, Shannon index) and beta (b, Bray Curtis distance) diversities of the gut microbiome between high VAT (*n* = 48) and low VAT (*n* = 51) individuals. Statistical significance of alpha and beta diversity was calculated by Wilcoxon-Mann-Whitney test and PERMANOVA with 999 permutations, respectively. Error bars represent the distribution of diversity scores. Numbers in graphs indicate P-values representing the difference of alpha and beta diversities between groups.

**Additional file 2: Supplementary Figure 2**. Profiling of the gut microbiome in human donors by BMI. a, A total of 99 human samples were included in the analysis and were divided by Body Mass Index (BMI). b,c, Alpha (b, Shannon index) and beta (c, Bray Curtis distance) diversities of the gut microbiome between obese (BMI ≥ 25, n=41) and normal individuals (BMI < 23, *n* = 54). Statistical significance of alpha and beta diversities was calculated by Wilcoxon-Mann-Whitney test and PERMANOVA with 999 permutations, respectively. Error bars represent the distribution of diversity scores. Numbers in graphs indicate P-values representing the difference of alpha and beta diversities between groups. d, A plot of linear discriminant analysis (LDA) scores from the linear discriminant analysis effect size (LEfSe) method illustrates the relative abundances of taxa that differ significantly between groups.

**Additional file 3: Supplementary Figure 3**. Profiling of the gut microbiome in human donors by WC. a, A total of 99 human samples were included in the analysis and were divided by waist circumference (WC). b,c, Alpha (b, Shannon index) and beta (c, Bray Curtis distance) diversities of the gut microbiome between high WC (*n* = 38) and low WC (*n* = 61) individuals. Statistical significance of alpha and beta diversities was calculated by Wilcoxon-Mann-Whitney test and PERMANOVA with 999 permutations, respectively. Error bars represent the distribution of diversity scores. Numbers in graphs indicate P-values representing the difference of alpha and beta diversities between groups. d, A taxonomic cladogram and a plot of linear discriminant analysis (LDA) scores from the linear discriminant analysis effect size (LEfSe) method illustrate the relative abundances of taxa that differ significantly between groups.

**Additional file 4: Supplementary Figure 4**. Profiling of the gut microbiome in human donors by serum marker. a, A total of 99 human samples were included in the analysis and were divided by blood TG levels (high TG, *n* = 25 and low TG, *n* = 74) or gamma glutamyl transpeptidase (γGTP) (high γGTP, *n* = 25 and low γGTP, *n* = 74). b,f, Alpha diversity of the gut microbiome between groups by (b), TG levels and (f), γGTP. c,g, Beta diversity of the gut microbiome between groups by (c), TG levels and (g), γGTP. Statistical significance of alpha and beta diversity were calculated by Wilcoxon-Mann-Whitney test and PERMANOVA with 999 permutations, respectively. Error bars represent the distribution of diversity scores. d,h, A plot of linear discriminant analysis (LDA) scores from the linear discriminant analysis effect size (LEfSe) method illustrates the relative abundances of taxa that differ significantly between groups by (d), TG levels and (h), γGTP. e, Boxplot is showing the relative abundance of *B. longum* between groups by TG levels*.* Numbers in graphs indicate P-values representing the difference of alpha and beta diversities between groups.

**Additional file 5: Supplementary Figure 5**. Profiling of the gut microbiome in human donors by fatty liver. a, A total of 99 human samples were included in the analysis and were divided by Incidence of fatty liver b,c, Alpha (b, Shannon index) and beta (c, Bray Curtis distance) diversities of the gut microbiome between groups with normal liver (*n* = 52) and fatty liver (*n* = 43). Error bars represent the distribution of diversity scores. Statistical significance of alpha and beta diversities was calculated by Wilcoxon-Mann-Whitney test and PERMANOVA with 999 permutations, respectively. Numbers in graphs indicate P-values representing the difference of alpha and beta diversities between groups. d, A plot of linear discriminant analysis (LDA) scores from the linear discriminant analysis effect size (LEfSe) method illustrates the relative abundances of taxa that differ significantly between groups.

**Additional file 6: Supplementary Figure 6**. Biological pathways enriched in effective microbiota to anti-obesity. a, Up-regulated pathways and DEGs in effective and non-effective strains of *B. longum* (same analysis to Fig. 2c)*.* Left panel: network representation of enriched Gene Ontology (GO) biological processes. Functionally related groups partially overlap. Right panel: volcano plot shows DEGs. b, Up-regulated pathways and DEGs in effective and non-effective strains of *B. bifidum* (same analysis to Fig. 2d)*.* Left panel: network representation of enriched Gene Ontology (GO) biological processes. Functionally related groups partially overlap. Statistical significance for pathway analysis was calculated using two-sided hypergeometric tests, and the false discovery rate was corrected using the Bonferroni step down method. Right panel: volcano plot shows DEGs. Red dots indicate genes with p-value < 0.05 and |log2 (fold change)| > 1.

**Additional file 7: Supplementary Figure 7**. Food intake and infiltrated-macrophage of HFD-fed mice with different treatment types. a, Each Vehicle, *B. longum_*MG732 and *B. bifidum_*MG731 was treated to HFD-fed mice and food intake was measured (*n* = 1-2 per group). b, CIBERSORT analysis of whole transcriptome obtained from gonadal white adipose tissue (gWAT) of HFD-fed mice treated with vehicle, *B. longum_*MG732 and *B. bifidum_*MG731.

**Additional file 8: Supplementary Figure 8**. Heatmap of GSEA from white adipose tissue and colon tissue. a, Heatmap for gene expression levels of gene set of oxidative phosphorylation, bile acid metabolism, inflammatory response, and cholesterol homeostasis in white adipose tissue from HFD-fed SPF mice. b, Heatmap for gene set of bile acid metabolism in colon tissue from HFD-fed SPF mice. Red and blue indicate expression levels above and below the median of each gene expression across the samples, respectively. The top 20 genes were shown.

**Additional file 9: Supplementary Figure 9**. Gene expression and metabolic markers in HFD-induced obesity mice treated with *B. longum*_Rosell 175. a, Gene expression profiles of thermogenesis and fatty acid oxidation in BAT b, Gene expression profiles involved in metabolism determined in liver. c, Metabolic marker level including hepatic TG, insulin, and cholesterol. (*n* = 5 per group). Statistical analysis was performed using one-way ANOVA with Tukey’s multiple comparison. Data expressed as mean ± S.E.M. For all graph, ns = non-significant.

**Additional file 10: Supplementary Figure 10**. Different microbial communities by inoculation of probiotics in germ-free mice. a,b, Beta (Bray-Curtis distance) diversities of the gut microbiome between (a), before inoculation of probiotics (0th week, *n* = 13) and after inoculation of probiotics (8th week, *n* = 15) in germ-free mice and (b), types of treatment (Pre, *n* = 13; HFD, *n* = 5; *B. longum*, *n* = 5, *B. bifidum*, *n* = 5). Each below table indicates statistical significance for beta diversities. Statistical significance of beta diversity was calculated by PERMANOVA with 999 permutations.

**Additional file 11: Supplementary Figure 11**. Heatmap of GSEA from colon tissue. Left panel: GSEA result obtained from colon tissue of HFD-fed GF mice. Right panel: Heatmap for gene set of cholesterol homeostasis in colon tissue from HFD-fed GF mice. Red and blue indicate expression levels above and below the median of each gene expression across the samples, respectively. The top 20 genes were shown.

**Additional file 12: Supplementary Figure 12**. Summary of mechanisms of *B. longum* and *B. bifidum* for anti-obesity in HFD-induced obesity mice. Inoculation of *B. longum* and *B. bifidum* induced physiological changes of WAT, BAT, and liver to prevent HFD-induced obesity in mice.

**Additional file 13: Supplementary Table 1**. Clinical characteristics of human donors.

**Additional file 14: Supplementary Table 2**. Primer list for qRT-PCR analysis.

## Data Availability

The metabolomic data reported in this paper have been deposited in the Mass Spectrometry Interactive Virtual Environment (accession no. MSV000088700). The sequence reported in this paper has been deposited in the European Nucleotide Archive (accession no. PRJEB33963).

## Abbreviations

HFD: High-fat diet
*B. longum*: Bifidobacterium longum
*B. bifidum*: Bifidobacterium bifidum
VAT: Visceral adipose tissue
BMI: Body mass index
WC: Waist circumstance
TG: Triglyceride
OXPHOS: Oxidative phosphorylation
SCFA: Short-chain fatty acid
BCAA: Branched-chain amino acid
IBD: Immune bowl disease
γGTP: Gamma-glutamyl transpeptidase
LDA: Linear discriminant analysis
ALT: Alanine aminotransferase
AST: Aspartate aminotransferase
