# Supplementary figures and images for: Bifidobacterial carbohydrate/nucleoside metabolism enhances oxidative phosphorylation in white adipose tissue to protect against diet-induced obesity
Source: Microbiome. 2022 Nov 4;10:188. doi: 10.1186/s40168-022-01374-0 (PMC9635107; doi:10.1186/s40168-022-01374-0)

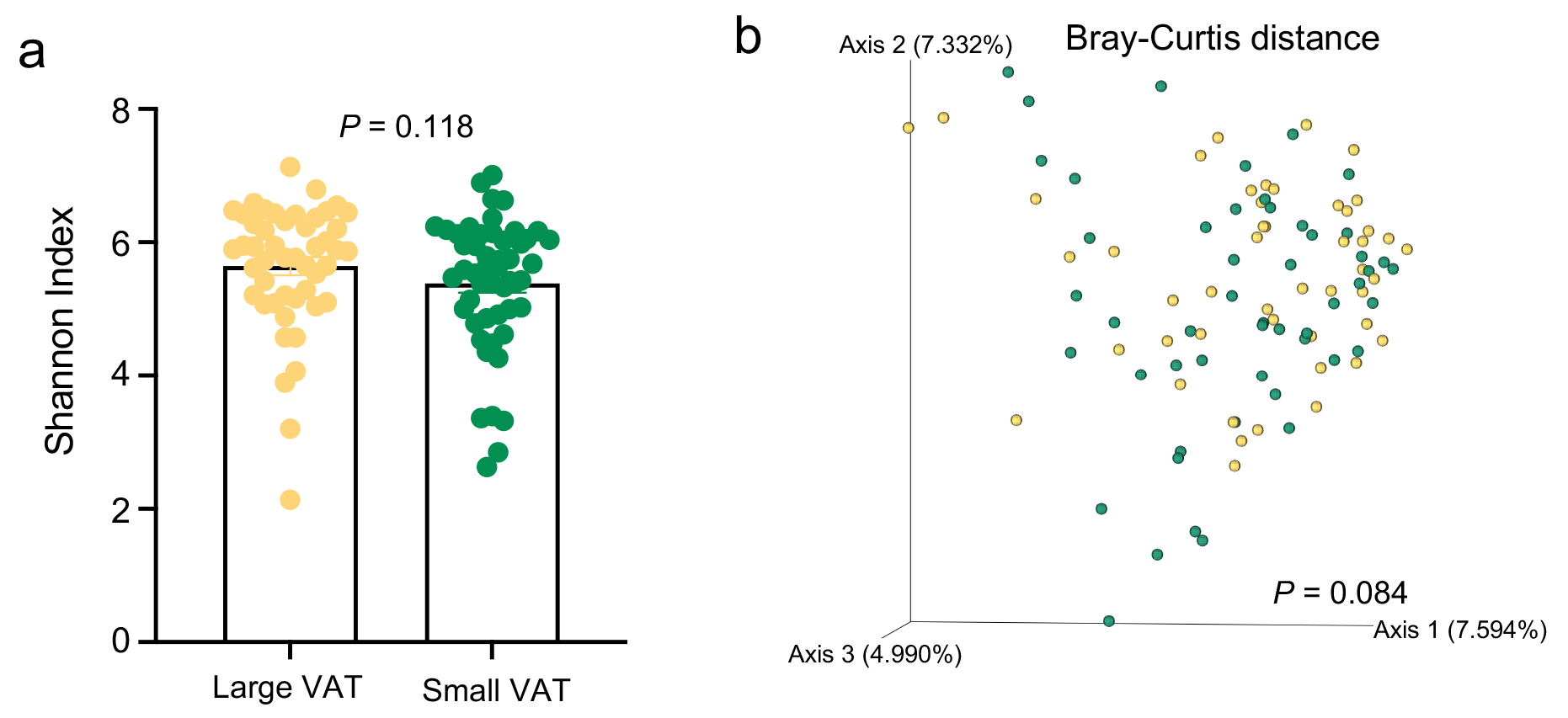

Supplement: Supplementary file 2 — Additional file 1: Supplementary Figure 1. Comparison of alpha and beta diversity of stool samples in human donors using visceral adipose tissue (VAT). a,b, Alpha (a, Shannon index) and beta (b, Bray Curtis distance) diversities of the gut microbiome between high VAT (n = 48) and low VAT (n = 51) individuals. Statistical significance of alpha and beta diversity was calculated by Wilcoxon-Mann-Whitney test and PERMANOVA with 999 permutations, respectively. Error bars represent the distribution of diversity scores. Numbers in graphs indicate P-values representing the difference of alpha and beta diversities between groups. [file 40168_2022_1374_MOESM1_ESM.tif]

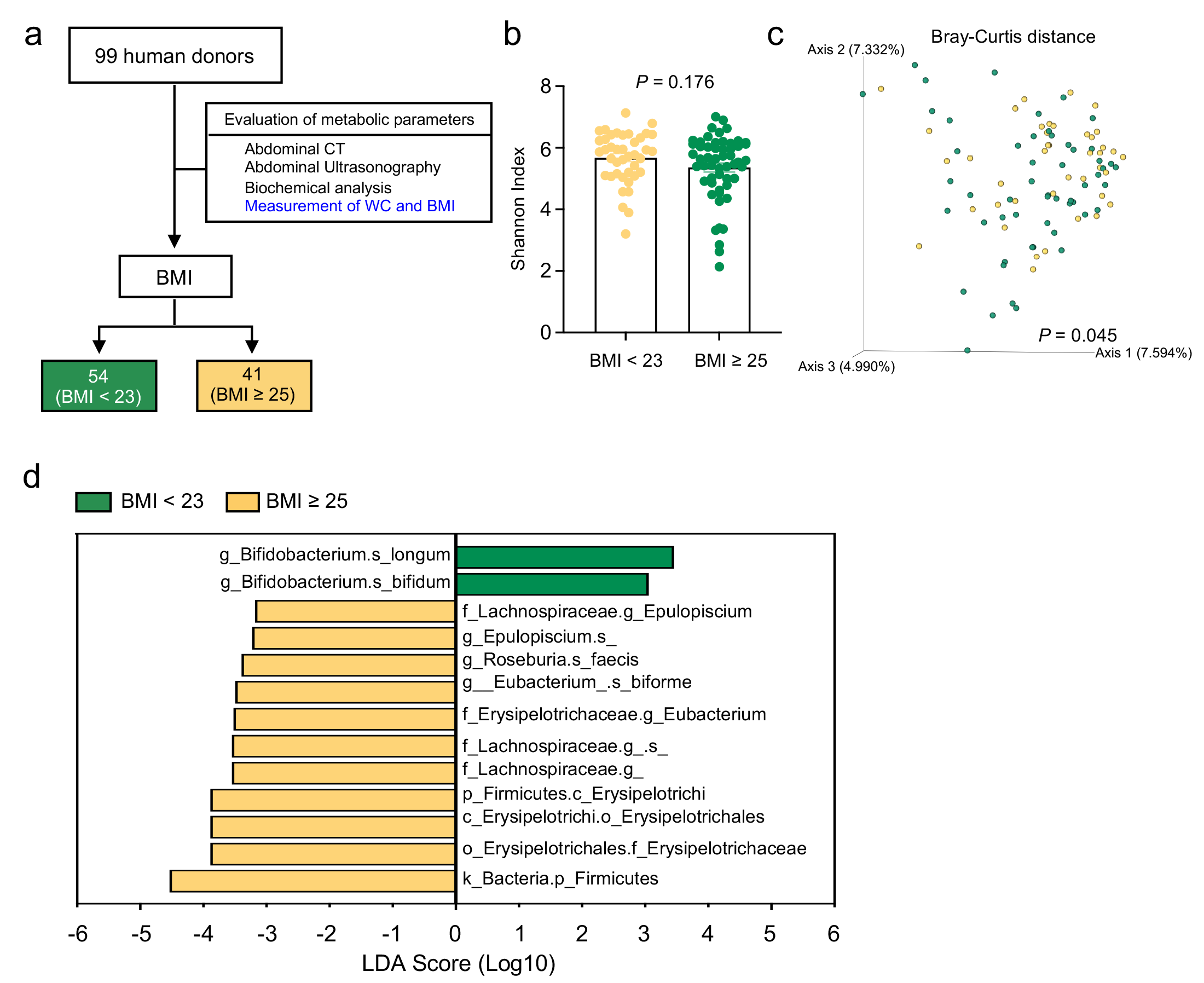

Supplement: Supplementary file 3 — Additional file 2: Supplementary Figure 2. Profiling of the gut microbiome in human donors by BMI. a, A total of 99 human samples were included in the analysis and were divided by Body Mass Index (BMI). b,c, Alpha (b, Shannon index) and beta (c, Bray Curtis distance) diversities of the gut microbiome between obese (BMI ≥ 25, n=41) and normal individuals (BMI < 23, n = 54). Statistical significance of alpha and beta diversities was calculated by Wilcoxon-Mann-Whitney test and PERMANOVA with 999 permutations, respectively. Error bars represent the distribution of diversity scores. Numbers in graphs indicate P-values representing the difference of alpha and beta diversities between groups. d, A plot of linear discriminant analysis (LDA) scores from the linear discriminant analysis effect size (LEfSe) method illustrates the relative abundances of taxa that differ significantly between groups. [file 40168_2022_1374_MOESM2_ESM.tif]

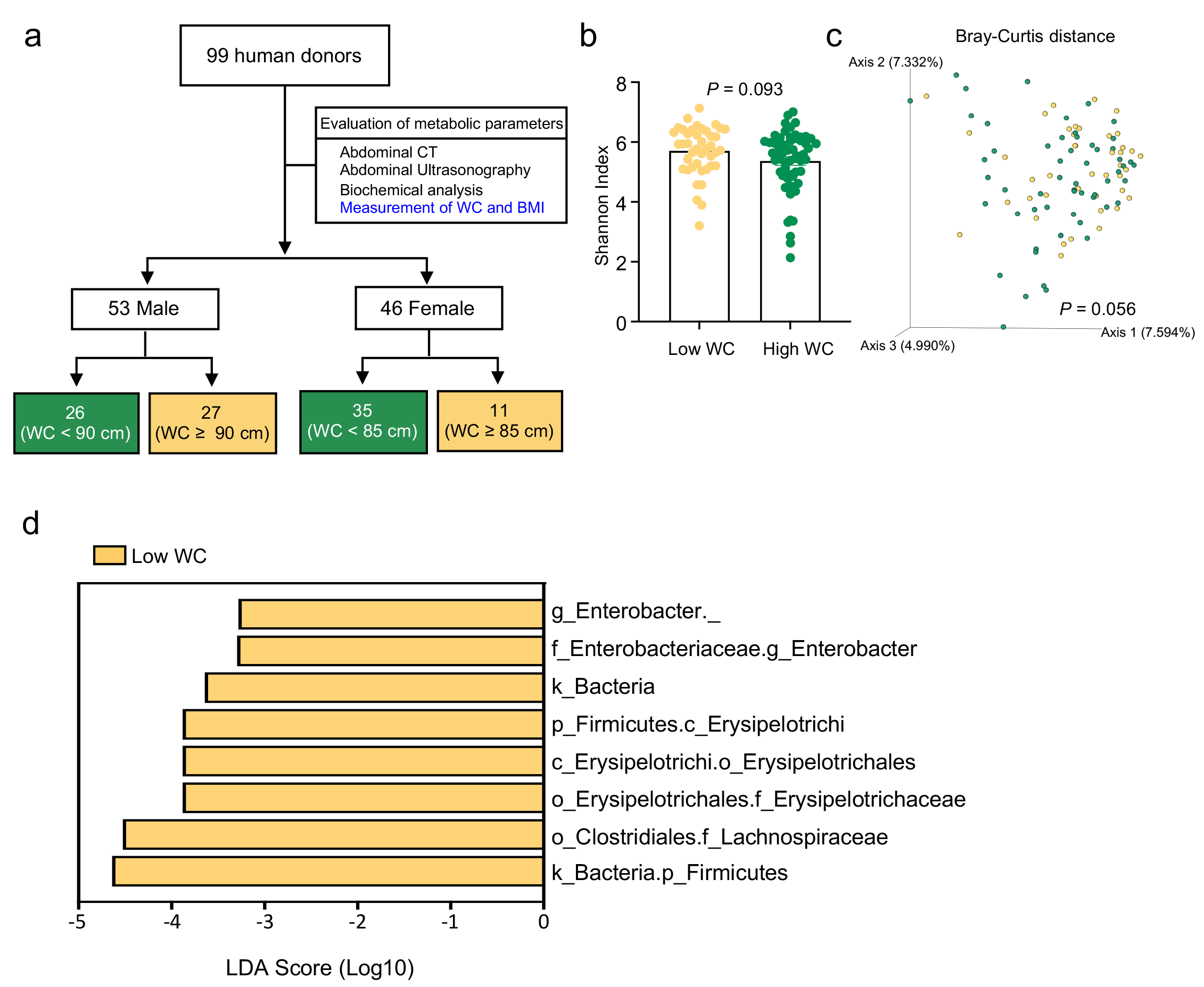

Supplement: Supplementary file 4 — Additional file 3: Supplementary Figure 3. Profiling of the gut microbiome in human donors by WC. a, A total of 99 human samples were included in the analysis and were divided by waist circumference (WC). b,c, Alpha (b, Shannon index) and beta (c, Bray Curtis distance) diversities of the gut microbiome between high WC (n = 38) and low WC (n = 61) individuals. Statistical significance of alpha and beta diversities was calculated by Wilcoxon-Mann-Whitney test and PERMANOVA with 999 permutations, respectively. Error bars represent the distribution of diversity scores. Numbers in graphs indicate P-values representing the difference of alpha and beta diversities between groups. d, A taxonomic cladogram and a plot of linear discriminant analysis (LDA) scores from the linear discriminant analysis effect size (LEfSe) method illustrate the relative abundances of taxa that differ significantly between groups. [file 40168_2022_1374_MOESM3_ESM.tif]

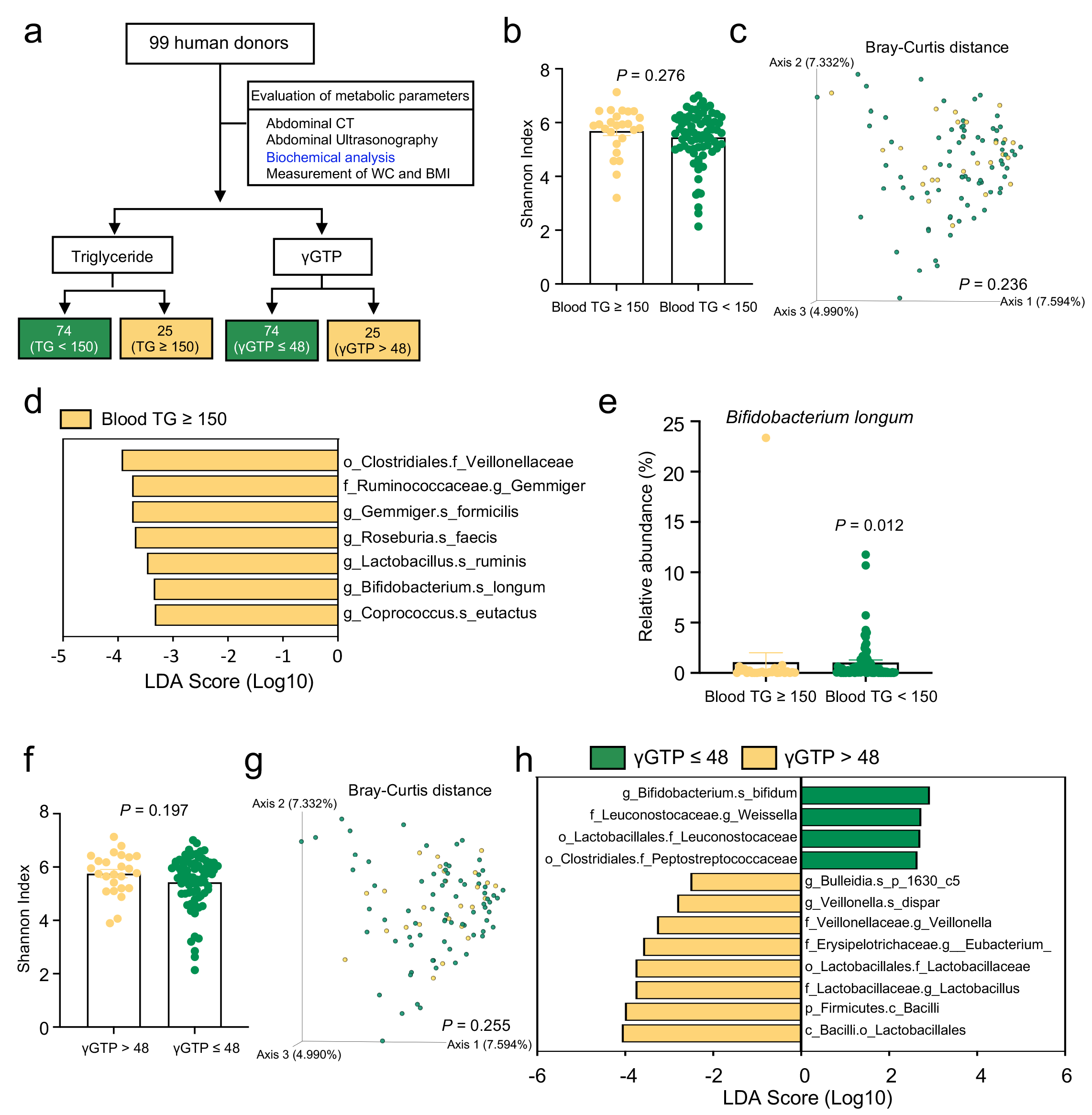

Supplement: Supplementary file 5 — Additional file 4: Supplementary Figure 4. Profiling of the gut microbiome in human donors by serum marker. a, A total of 99 human samples were included in the analysis and were divided by blood TG levels (high TG, n = 25 and low TG, n = 74) or gamma glutamyl transpeptidase (γGTP) (high γGTP, n = 25 and low γGTP, n = 74). b,f, Alpha diversity of the gut microbiome between groups by (b), TG levels and (f), γGTP. c,g, Beta diversity of the gut microbiome between groups by (c), TG levels and (g), γGTP. Statistical significance of alpha and beta diversity were calculated by Wilcoxon-Mann-Whitney test and PERMANOVA with 999 permutations, respectively. Error bars represent the distribution of diversity scores. d,h, A plot of linear discriminant analysis (LDA) scores from the linear discriminant analysis effect size (LEfSe) method illustrates the relative abundances of taxa that differ significantly between groups by (d), TG levels and (h), γGTP. e, Boxplot is showing the relative abundance of B. longum between groups by TG levels. Numbers in graphs indicate P-values representing the difference of alpha and beta diversities between groups. [file 40168_2022_1374_MOESM4_ESM.tif]

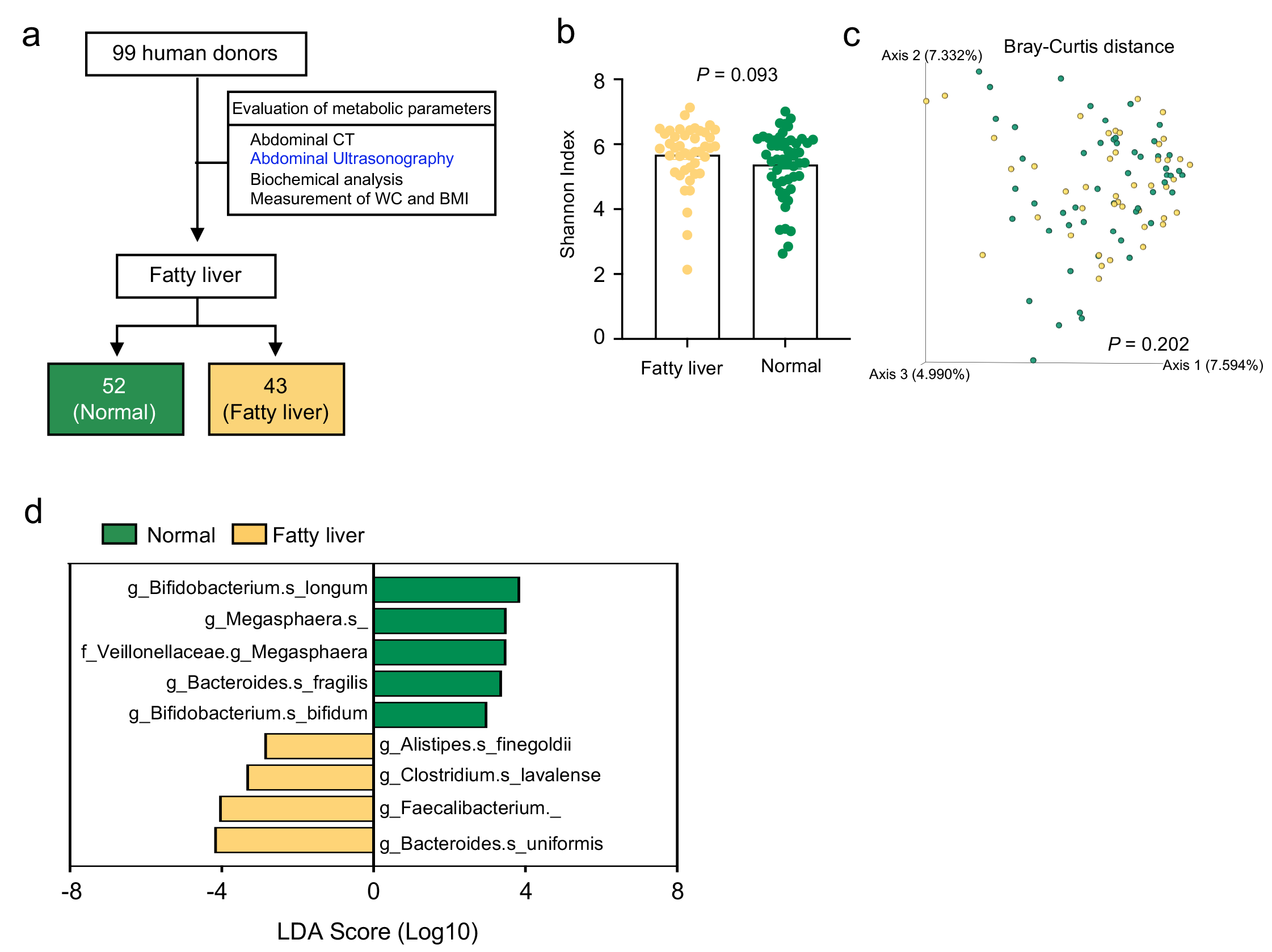

Supplement: Supplementary file 6 — Additional file 5: Supplementary Figure 5. Profiling of the gut microbiome in human donors by fatty liver. a, A total of 99 human samples were included in the analysis and were divided by Incidence of fatty liver b,c, Alpha (b, Shannon index) and beta (c, Bray Curtis distance) diversities of the gut microbiome between groups with normal liver (n = 52) and fatty liver (n = 43). Error bars represent the distribution of diversity scores. Statistical significance of alpha and beta diversities was calculated by Wilcoxon-Mann-Whitney test and PERMANOVA with 999 permutations, respectively. Numbers in graphs indicate P-values representing the difference of alpha and beta diversities between groups. d, A plot of linear discriminant analysis (LDA) scores from the linear discriminant analysis effect size (LEfSe) method illustrates the relative abundances of taxa that differ significantly between groups. [file 40168_2022_1374_MOESM5_ESM.tif]

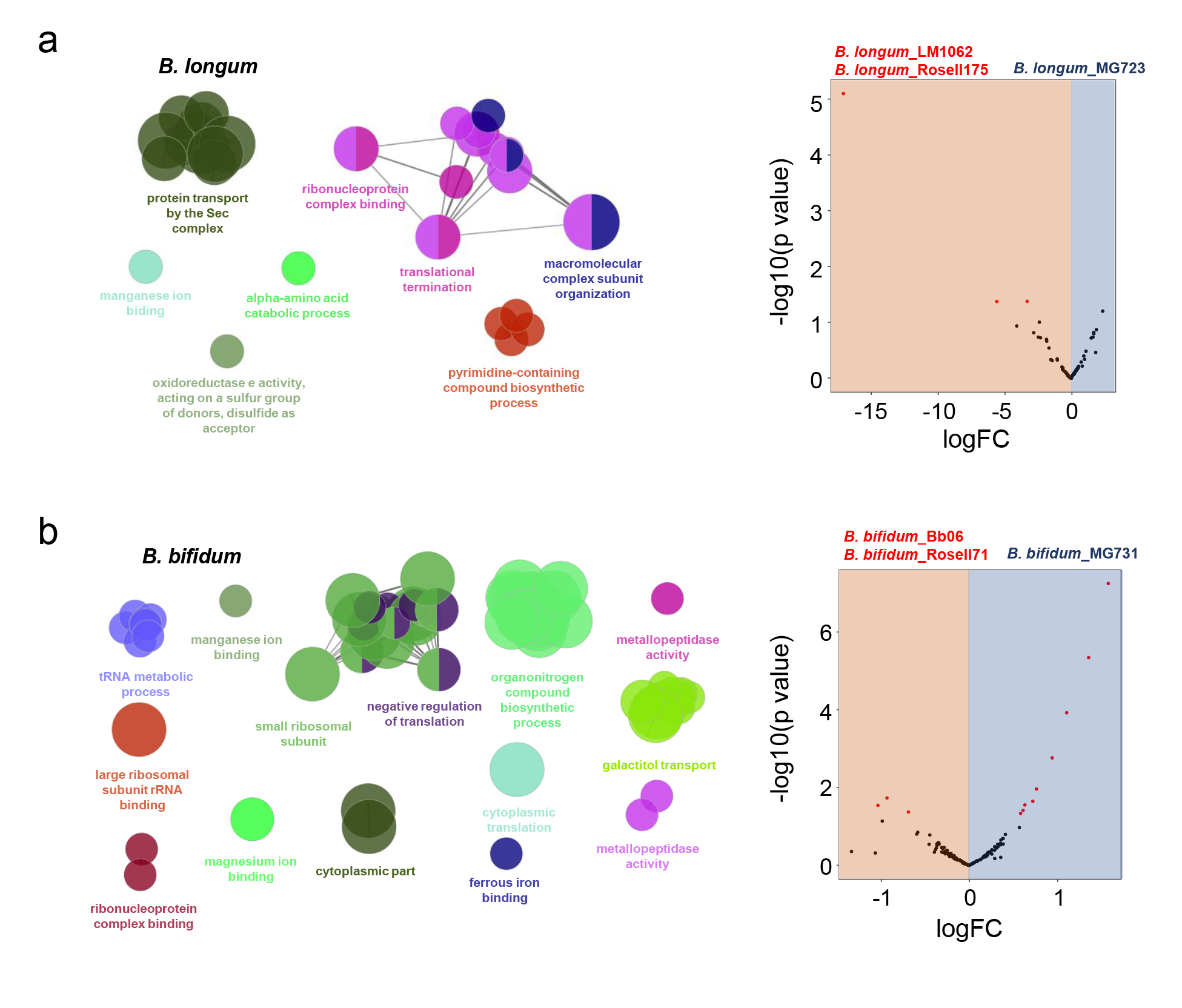

Supplement: Supplementary file 7 — Additional file 6: Supplementary Figure 6. Biological pathways enriched in effective microbiota to anti-obesity. a, Up-regulated pathways and DEGs in effective and non-effective strains of B. longum (same analysis to Fig. 2c). Left panel: network representation of enriched Gene Ontology (GO) biological processes. Functionally related groups partially overlap. Right panel: volcano plot shows DEGs. b, Up-regulated pathways and DEGs in effective and non-effective strains of B. bifidum (same analysis to Fig. 2d). Left panel: network representation of enriched Gene Ontology (GO) biological processes. Functionally related groups partially overlap. Statistical significance for pathway analysis was calculated using two-sided hypergeometric tests, and the false discovery rate was corrected using the Bonferroni step down method. Right panel: volcano plot shows DEGs. Red dots indicate genes with p-value < 0.05 and |log2 (fold change)| > 1. [file 40168_2022_1374_MOESM6_ESM.tif]

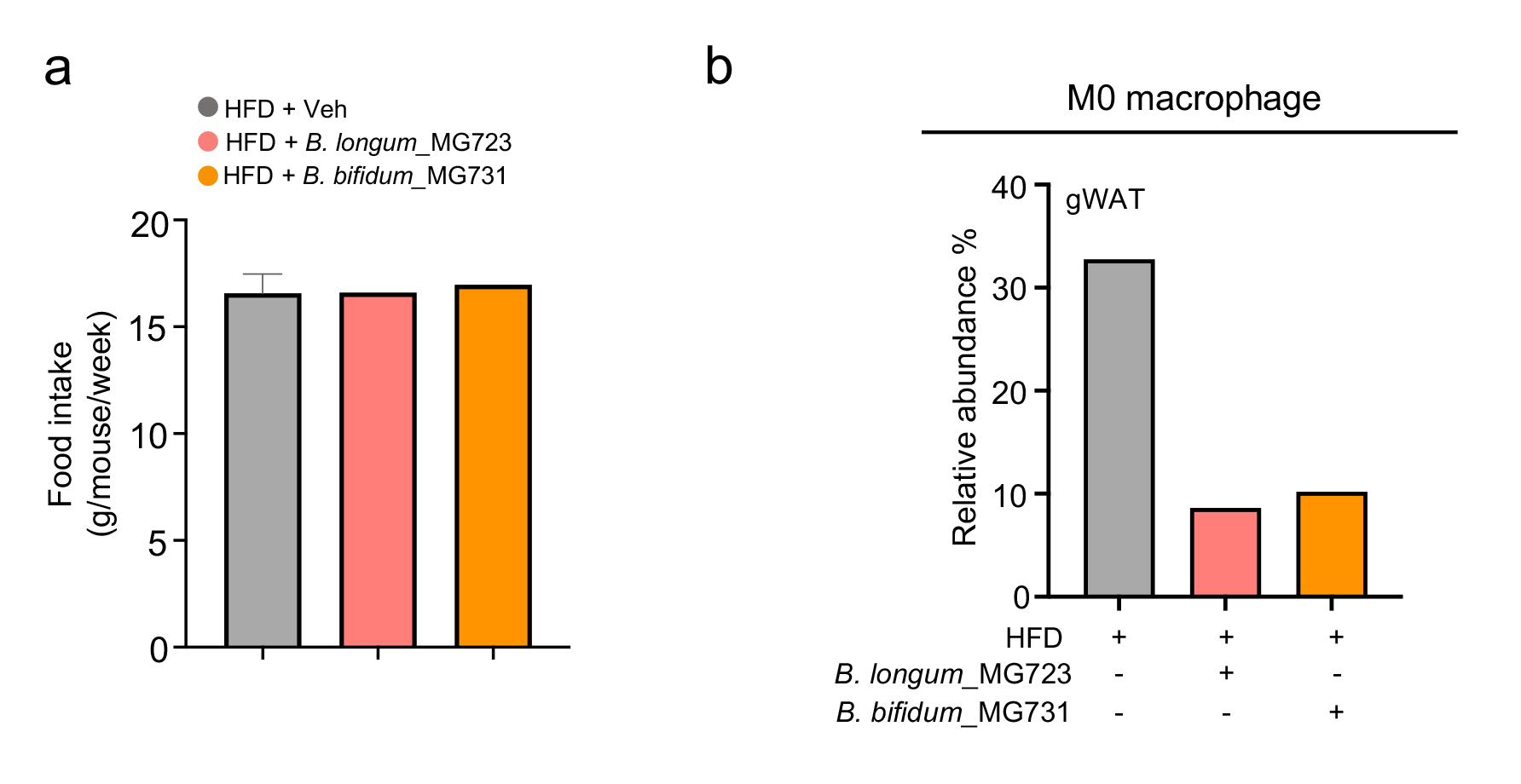

Supplement: Supplementary file 8 — Additional file 7: Supplementary Figure 7. Food intake and infiltrated-macrophage of HFD-fed mice with different treatment types. a, Each Vehicle, B. longum_MG732 and B. bifidum_MG731 was treated to HFD-fed mice and food intake was measured (n = 1-2 per group). b, CIBERSORT analysis of whole transcriptome obtained from gonadal white adipose tissue (gWAT) of HFD-fed mice treated with vehicle, B. longum_MG732 and B. bifidum_MG731. [file 40168_2022_1374_MOESM7_ESM.tif]

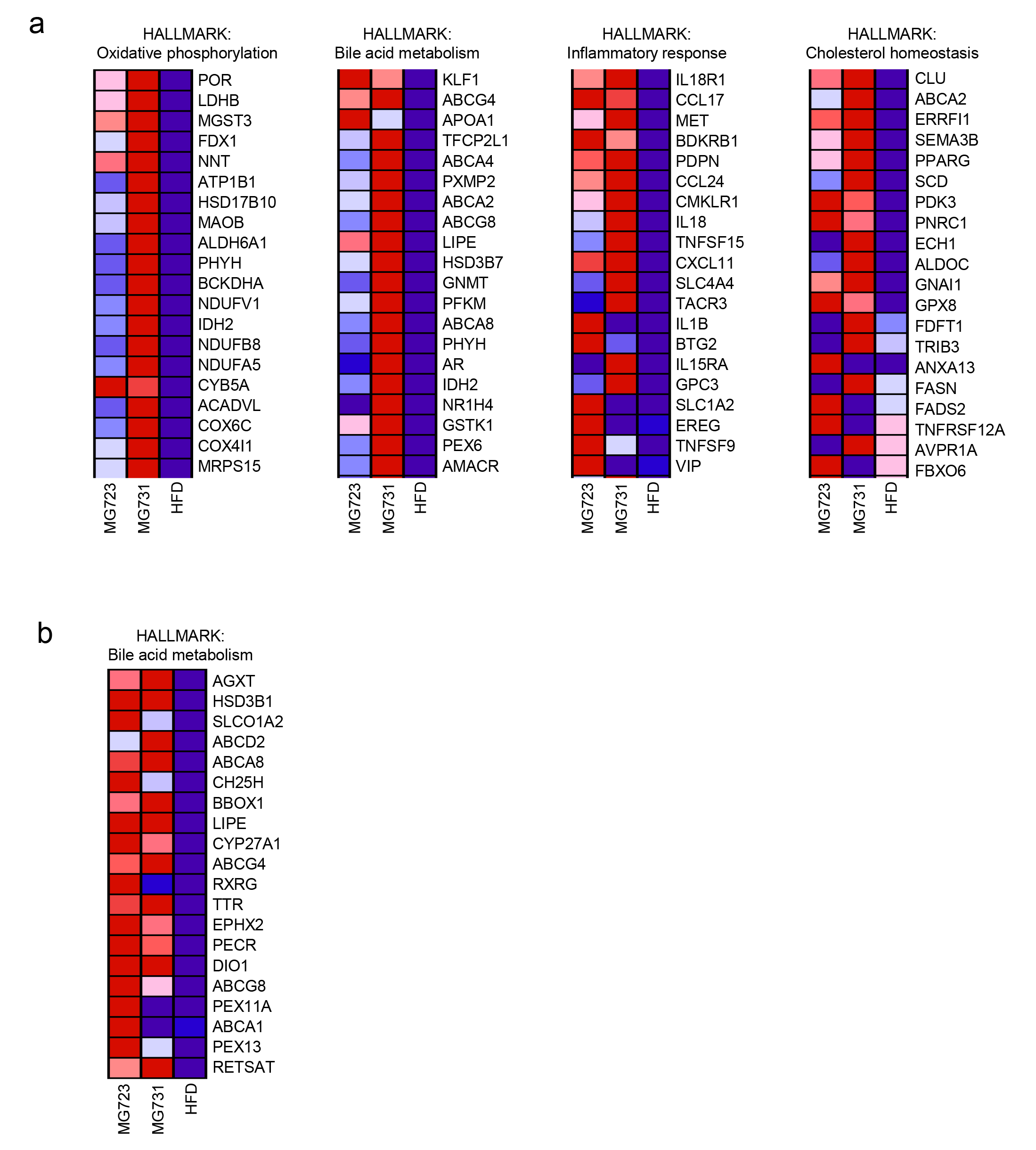

Supplement: Supplementary file 9 — Additional file 8: Supplementary Figure 8. Heatmap of GSEA from white adipose tissue and colon tissue. a, Heatmap for gene expression levels of gene set of oxidative phosphorylation, bile acid metabolism, inflammatory response, and cholesterol homeostasis in white adipose tissue from HFD-fed SPF mice. b, Heatmap for gene set of bile acid metabolism in colon tissue from HFD-fed SPF mice. Red and blue indicate expression levels above and below the median of each gene expression across the samples, respectively. The top 20 genes were shown. [file 40168_2022_1374_MOESM8_ESM.tif]

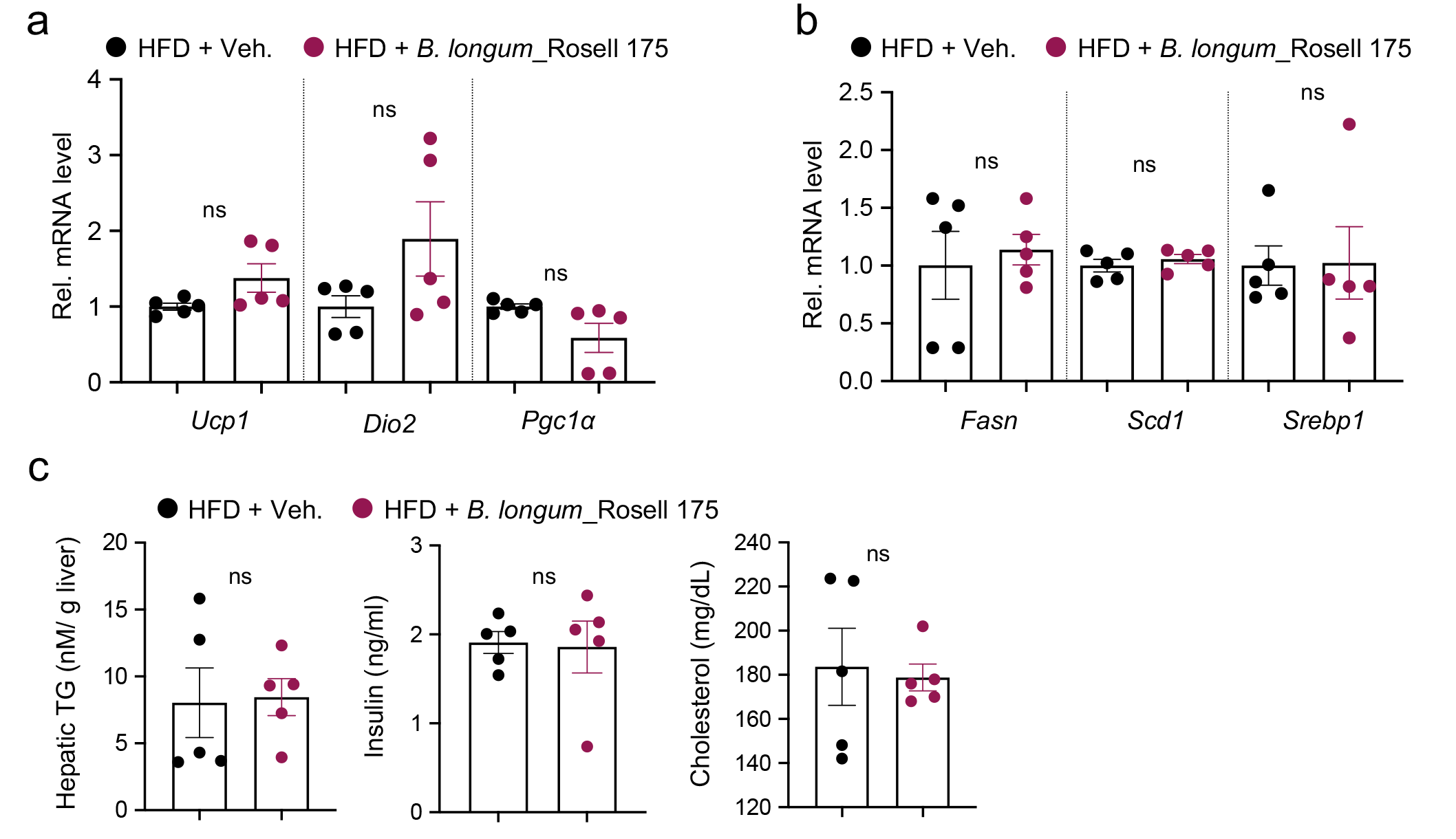

Supplement: Supplementary file 10 — Additional file 9: Supplementary Figure 9. Gene expression and metabolic markers in HFD-induced obesity mice treated with B. longum_Rosell 175. a, Gene expression profiles of thermogenesis and fatty acid oxidation in BAT b, Gene expression profiles involved in metabolism determined in liver. c, Metabolic marker level including hepatic TG, insulin, and cholesterol. (n = 5 per group). Statistical analysis was performed using one-way ANOVA with Tukey’s multiple comparison. Data expressed as mean ± S.E.M. For all graph, ns = non-significant. [file 40168_2022_1374_MOESM9_ESM.tif]

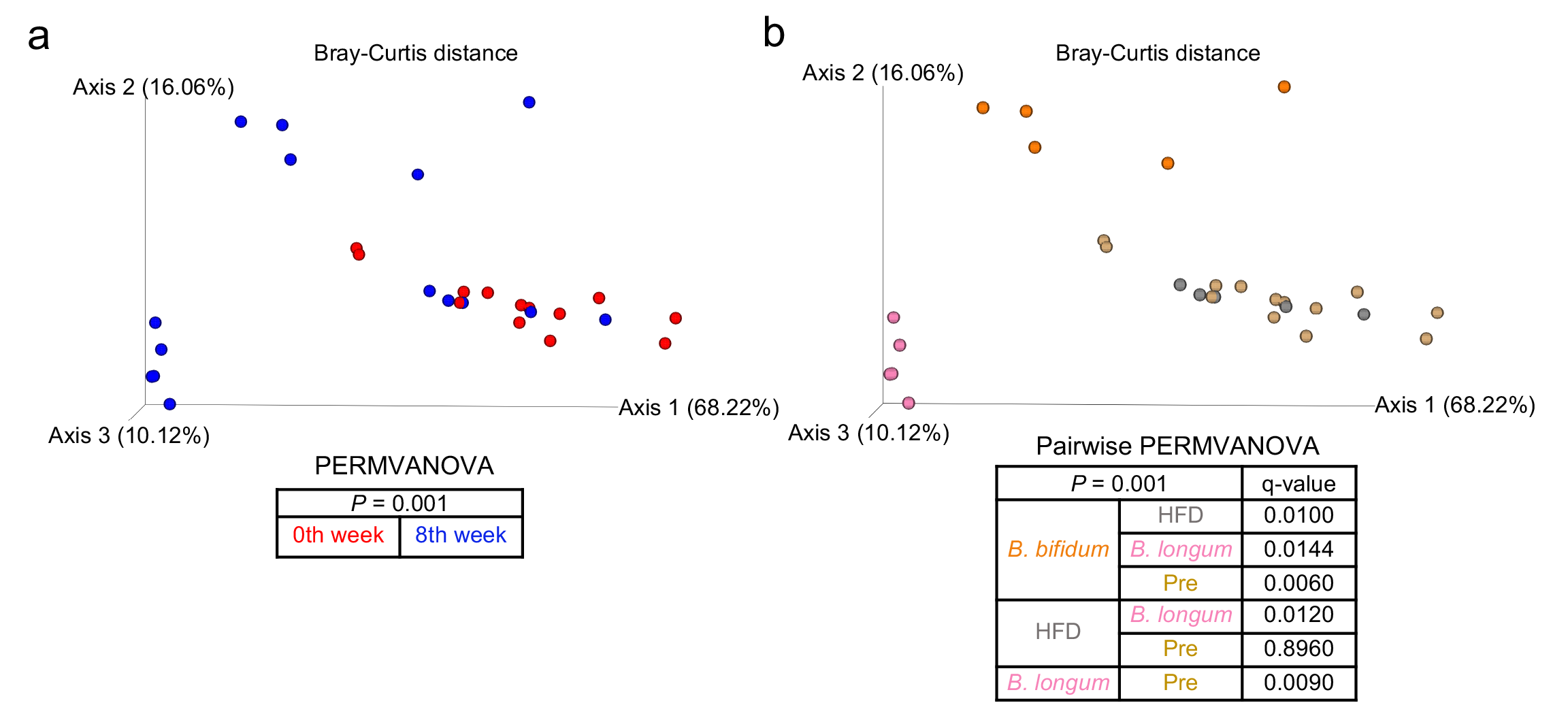

Supplement: Supplementary file 11 — Additional file 10: Supplementary Figure 10. Different microbial communities by inoculation of probiotics in germ-free mice. a,b, Beta (Bray-Curtis distance) diversities of the gut microbiome between (a), before inoculation of probiotics (0th week, n = 13) and after inoculation of probiotics (8th week, n = 15) in germ-free mice and (b), types of treatment (Pre, n = 13; HFD, n = 5; B. longum, n = 5, B. bifidum, n = 5). Each below table indicates statistical significance for beta diversities. Statistical significance of beta diversity was calculated by PERMANOVA with 999 permutations. [file 40168_2022_1374_MOESM10_ESM.tif]

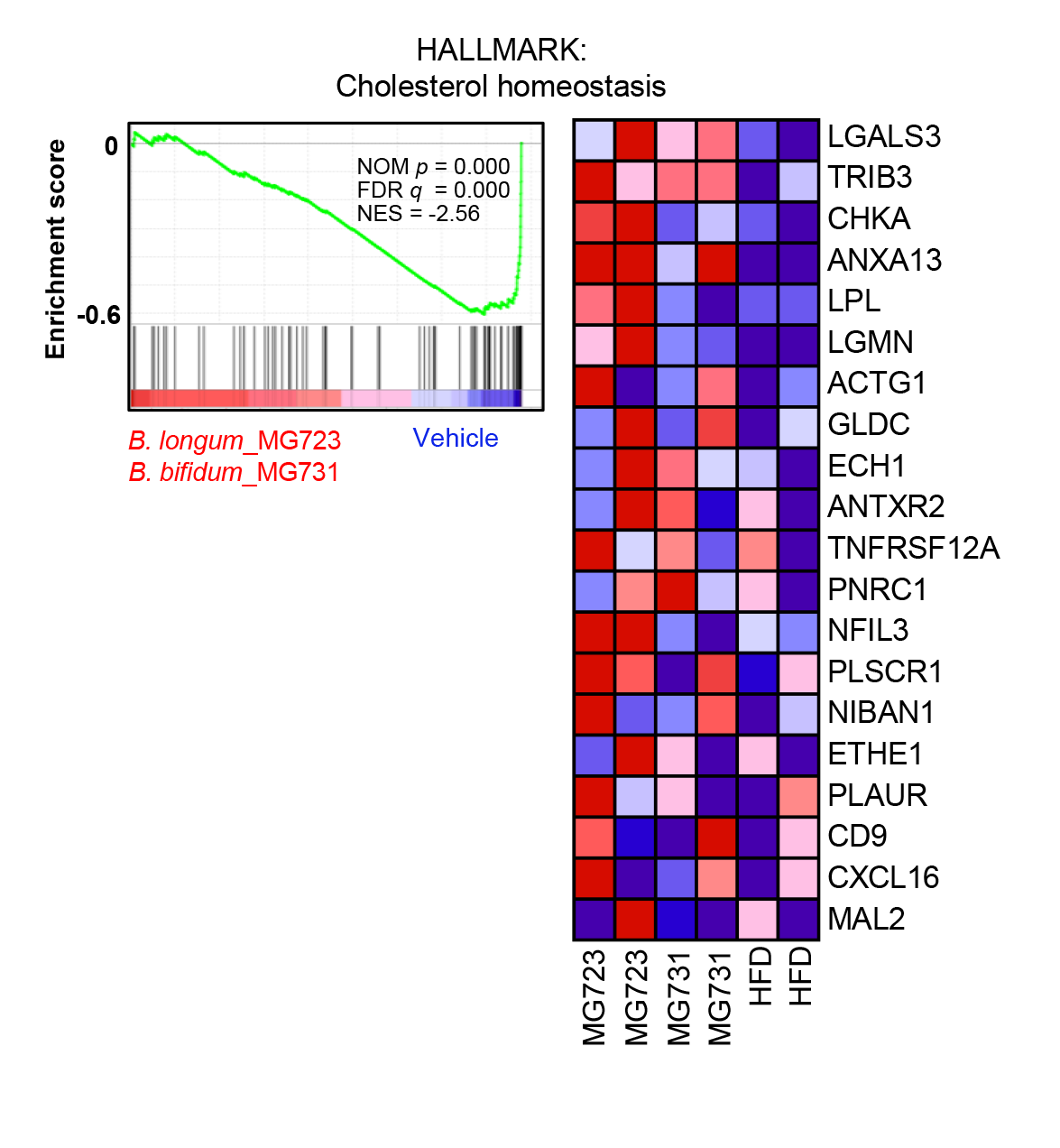

Supplement: Supplementary file 12 — Additional file 11: Supplementary Figure 11. Heatmap of GSEA from colon tissue. Left panel: GSEA result obtained from colon tissue of HFD-fed GF mice. Right panel: Heatmap for gene set of cholesterol homeostasis in colon tissue from HFD-fed GF mice. Red and blue indicate expression levels above and below the median of each gene expression across the samples, respectively. The top 20 genes were shown. [file 40168_2022_1374_MOESM11_ESM.tif]

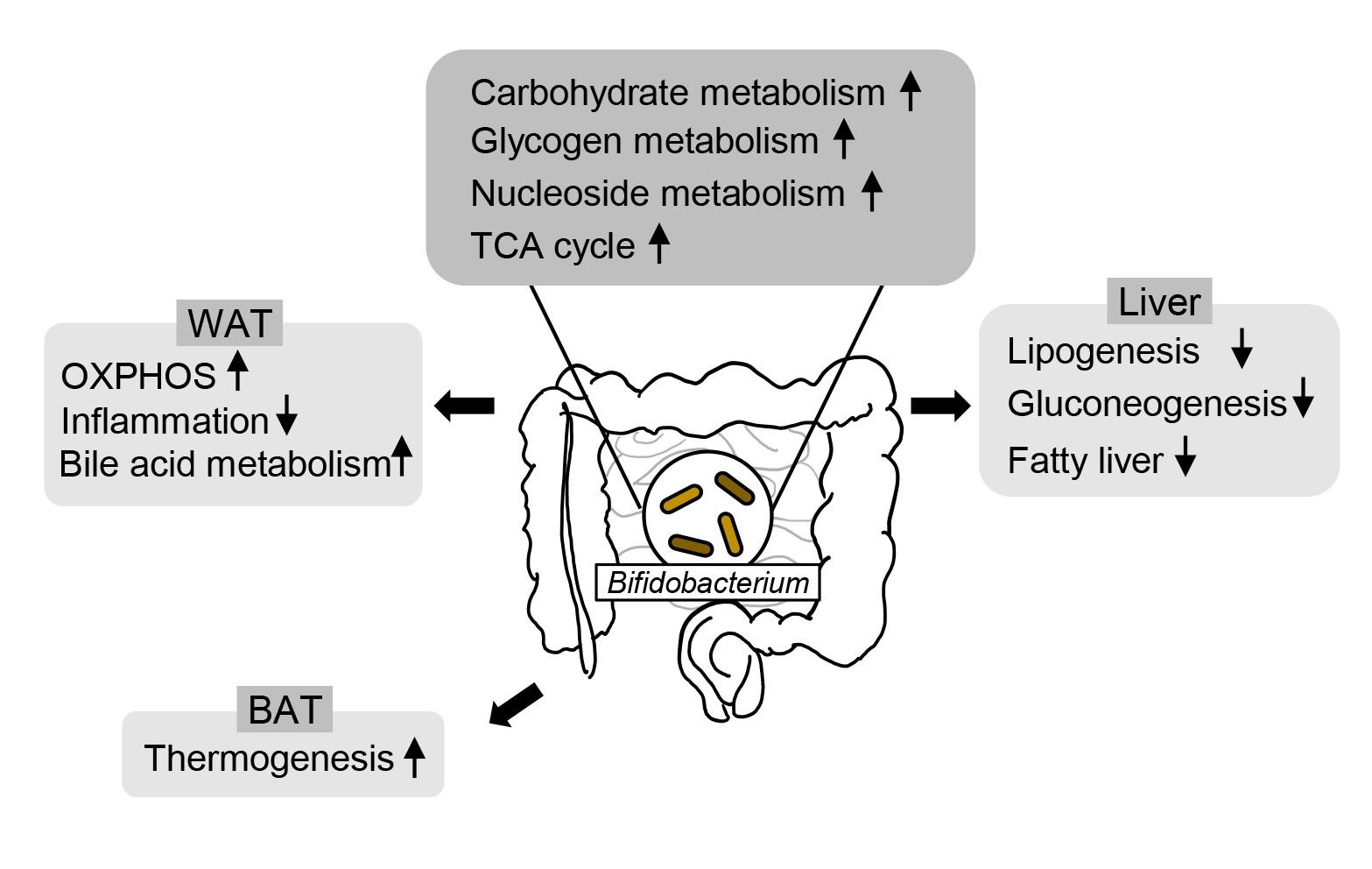

Supplement: Supplementary file 13 — Additional file 12: Supplementary Figure 12. Summary of mechanisms of B. longum and B. bifidum for anti-obesity in HFD-induced obesity mice. Inoculation of B. longum and B. bifidum induced physiological changes of WAT, BAT, and liver to prevent HFD-induced obesity in mice. [file 40168_2022_1374_MOESM12_ESM.tif]
