# Supplementary material for: Bifidobacterial carbohydrate/nucleoside metabolism enhances oxidative phosphorylation in white adipose tissue to protect against diet-induced obesity
Source: Microbiome. 2022 Nov 4;10:188. doi: 10.1186/s40168-022-01374-0 (PMC9635107; doi:10.1186/s40168-022-01374-0)
Supplement: Supplementary file 14 — Additional file 13: Supplementary Table 1. Clinical characteristics of human donors. [file 40168_2022_1374_MOESM13_ESM.pdf]

SampleID	Sex	Age	Height	Weight	BMI	BMI_categorical	VAT	VAT_categorical	WC	WC_categorical	FAT_liver	TG	TG_categorical	CHOLESTEROL	GGT	GGT_categorical
GS084	F	42	157.4	53.4	21.5541954	Low	24.7	Low	70.5	Low	Normal_liver	52	Low	202	11	Low
GS024	M	57	169.8	62.3	21.6079063	Low	58.6	Low	82	Low	Normal_liver	126	Low	184	12	Low
GS100	F	36	161.8	55.9	21.3527966	Low	60.8	Low	72	Low	Normal_liver	84	Low	190	12	Low
GS103	F	51	162.2	58.9	22.3879128	Low	42.8	Low	79	Low	Normal_liver	60	Low	159	12	Low
GS117	F	57	152.3	47.6	20.5214081	Low	26.5	Low	68.5	Low	Normal_liver	69	Low	238	12	Low
GS135	F	55	156.4	52.6	21.5036526	Low	65.5	Low	77	Low	Normal_liver	54	Low	210	12	Low
GS029	F	59	153	43.5	18.5825958	Low	36.4	Low	67	Low	Normal_liver	50	Low	206	13	Low
GS052	M	50	174.8	61.2	20.0294285	Low	45.1	Low	78	Low	Normal_liver	45	Low	169	13	Low
GS058	F	46	154.2	50.2	21.1122723	Low	16.8	Low	75	Low	Normal_liver	56	Low	254	13	Low
GS065	M	47	167.8	61	21.664362	Low	87.6	Low	81	Low	Normal_liver	148	Low	175	13	Low
GS142	F	61	148.4	44.8	20.3427753	Low	34.5	Low	67.5	Low	Normal_liver	65	Low	216	13	Low
GS148	M	49	174.9	56.8	18.5681534	Low	34.5	Low	70.5	Low	Fatty_liver	63	Low	142	13	Low
GS162	F	52	156.1	55.9	22.9406643	Low	52.2	Low	78	Low	Normal_liver	33	Low	235	13	Low
GS010	F	61	154.1	52.1	21.9397888	Low	69.5	Low	80.5	Low	Normal_liver	81	Low	174	14	Low
GS041	F	62	164.2	60.1	22.2909298	Low	26.1	Low	83	Low	Normal_liver	114	Low	256	14	Low
GS094	F	49	167.2	58.5	20.9258595	Low	63.4	Low	81.5	Low	Normal_liver	63	Low	187	14	Low
GS011	M	61	172.5	61.8	20.7687454	Low	33.5	Low	78.5	Low	Normal_liver	64	Low	179	15	Low
GS017	F	64	147.5	48.6	22.3384094	Low	32.2	Low	75.5	Low	Normal_liver	89	Low	263	15	Low
GS081	F	47	156	55.9	22.9700851	Low	44	Low	80	Low	Normal_liver	50	Low	192	15	Low
GS116	F	57	155.7	49.7	20.501194	Low	45.7	Low	73	Low	Normal_liver	49	Low	199	15	Low
GS122	F	45	166.8	61.9	22.2483883	Low	57.2	Low	82	Low	Normal_liver	42	Low	220	15	Low
GS123	M	64	167.9	54.7	19.4037609	Low	82.2	Low	77.5	Low	Normal_liver	54	Low	222	15	Low
GS125	F	42	154.1	45.8	19.2868004	Low	51.6	Low	77	Low	Normal_liver	57	Low	202	15	Low
GS056	F	56	155.5	47.2	19.5200634	Low	59.2	Low	68	Low	Normal_liver	50	Low	230	17	Low
GS154	M	65	165.3	60.4	22.1050358	Low	73.1	Low	80	Low	Normal_liver	67	Low	153	17	Low
GS031	F	64	162.7	57.1	21.5705395	Low	55.3	Low	83	Low	Normal_liver	70	Low	213	18	Low
GS140	F	46	149.8	45	20.0534401	Low	62	Low	72	Low	Normal_liver	68	Low	226	18	Low
GS001	F	63	155.8	49.9	20.5572796	Low	51.5	Low	72	Low	Normal_liver	92	Low	171	20	Low
GS086	M	57	175.5	95.4	30.973774	High	282	High	112	High	Fatty_liver	41	Low	167	20	Low
GS088	M	57	174	79.5	26.2584229	High	244.9	High	101.5	High	Fatty_liver	122	Low	158	20	Low
GS095	M	58	166.9	58	20.8216591	Low	65.3	Low	78	Low	Normal_liver	92	Low	249	20	Low
GS113	M	45	188	69.3	19.6072884	Low	67.7	Low	76.5	Low	Normal_liver	123	Low	171	20	Low
GS120	F	64	145.8	40.5	19.0519733	Low	26.7	Low	73	Low	Normal_liver	152	High	266	20	Low
GS158	F	55	155.6	51.8	21.3949146	Low	63.3	Low	75	Low	Normal_liver	66	Low	185	20	Low
GS025	M	60	160.7	68	26.3315945	High	243.7	High	89	High	Fatty_liver	86	Low	158	21	Low
GS032	M	58	170.4	61.4	21.1460476	Low	55.3	Low	76	Low		111	Low	228	21	Low
GS042	F	60	161.3	75.4	28.9802818	High	184.2	High	101.5	High		123	Low	213	21	Low
GS070	F	64	143.3	54.3	26.4428005	High	152.2	High	83	Low	Fatty_liver	90	Low	233	21	Low
GS107	F	58	146.4	46.8	21.8355274	Low	155.6	High	80	Low	Fatty_liver	99	Low	211	21	Low
GS022	F	56	145.8	52.9	24.88517	Normal	169.7	High	83.5	Low	Fatty_liver	366	High	227	22	Low
GS055	M	62	175.1	60.2	19.634697	Low	93.3	Low	78	Low	Normal_liver	91	Low	176	22	Low
GS082	F	62	161.2	66.2	25.4758053	High	156.9	High	91	High	Fatty_liver	105	Low	238	22	Low
GS165	M	47	172.5	66.5	22.3482456	Low	76.3	Low	87	Low	Normal_liver	52	Low	211	22	Low
GS092	F	52	157.2	50.4	20.3950825	Low	33.3	Low	75	Low	Normal_liver	117	Low	170	23	Low
GS037	M	60	167.7	74.1	26.3482685	High	240.5	High	90	High	Normal_liver	83	Low	173	24	Low
GS040	F	45	160.9	51.2	19.7768841	Low	41.7	Low	66.5	Low	Normal_liver	82	Low	143	24	Low
GS062	F	45	156.7	48.4	19.7109413	Low	27.1	Low	70.5	Low	Normal_liver	66	Low	192	24	Low
GS097	F	47	152.9	53.5	22.8843613	Low	54.5	Low	73.5	Low	Normal_liver	55	Low	163	24	Low
GS139	F	61	159.7	57.8	22.6630325	Low	169	High	84.5	Low	Fatty_liver	135	Low	212	24	Low
GS036	F	58	156.9	56.5	22.9510498	Low	153	High	87	High	Fatty_liver	125	Low	184	25	Low
GS049	F	61	152.6	53.4	22.9314861	Low	53.8	Low	81.5	Low	Normal_liver	64	Low	210	25	Low
GS101	M	55	179.1	69.4	21.6355686	Low	90.6	Low	85.5	Low	Normal_liver	147	Low	235	25	Low
GS111	F	37	158.5	55.8	22.2113857	Low	69	Low	82	Low	Fatty_liver	95	Low	217	25	Low
GS083	M	52	175.9	76.3	24.6599884	Normal	234.8	High	93.5	High	Fatty_liver	113	Low	204	27	Low
GS114	M	61	156.8	54	21.9635048	Low	87.4	Low	82	Low	Normal_liver	87	Low	200	27	Low
GS075	M	41	182.4	71.6	21.5210457	Low	43.3	Low	78	Low	Normal_liver	35	Low	209	28	Low
GS132	F	40	166.9	82.4	29.5811157	High	177.1	High	93.5	High	Fatty_liver	229	High	301	28	Low
GS006	F	60	153.8	93.8	39.6542893	High	222.3	High	110	High	Fatty_liver	442	High	206	29	Low
GS150	M	31	179.1	66.7	20.7938385	Low	76.8	Low	79	Low		56	Low	223	29	Low
GS155	M	65	162.7	83.6	31.5813847	High	263	High	102	High	Fatty_liver	158	High	208	30	Low
GS118	M	57	164.6	62.1	22.9209137	Low	47.2	Low	86	Low	Normal_liver	36	Low	209	31	Low
GS157	F	65	148	69.1	31.5467491	High	208.6	High	103	High	Fatty_liver	137	Low	253	32	Low
GS015	M	51	173.4	76.9	25.575737	High	218.2	High	92	High		101	Low	216	33	Low
GS136	F	64	156.1	58.3	23.9255943	Normal	155.6	High	88.5	High	Fatty_liver	47	Low	137	33	Low
GS007	F	49	162.5	54.8	20.7526627	Low	58.1	Low	69	Low	Normal_liver	88	Low	158	34	Low
GS033	M	65	174.1	82.4	27.1850205	High	246.6	High	98	High	Fatty_liver	91	Low	176	36	Low
GS099	M	60	169.5	61.2	21.30159	Low	75.9	Low	81	Low	Fatty_liver	78	Low	102	36	Low
GS124	F	58	162.7	68.1	25.7259846	High	211.8	High	89.5	High	Fatty_liver	275	High	229	37	Low
GS073	M	62	168.9	71.4	25.0287361	High	202.5	High	88	Low	Fatty_liver	121	Low	168	41	Low
GS161	M	60	159.3	72.4	28.530344	High	203.1	High	90	High	Fatty_liver	234	High	210	42	Low
GS091	M	58	179.6	84.1	26.0725384	High	254.6	High	95.5	High	Fatty_liver	167	High	197	44	Low
GS121	M	63	172.3	90.6	30.5181103	High	246	High	105	High	Fatty_liver	232	High	189	44	Low
GS068	M	63	181.4	74.2	22.5491066	Low	40.7	Low	85	Low	Normal_liver	54	Low	216	45	Low
GS144	F	58	161.6	70	26.8049698	High	191.6	High	97	High	Fatty_liver	91	Low	368	45	Low
GS093	M	64	162.4	68.1	25.8211193	High	201.5	High	91	Low	Fatty_liver	190	High	220	49	High
GS128	M	39	177.8	92.2	29.1653652	High	205.5	High	106.5	High	Fatty_liver	124	Low	155	49	High
GS074	M	51	172.3	86.3	29.0696793	High	207	High	96	High	Fatty_liver	195	High	225	50	High
GS016	M	50	172.2	81.2	27.3835487	High	235.9	High	98.5	High	Fatty_liver	583	High	203	52	High
GS133	M	55	178.8	86.4	27.0258102	High	232.7	High	94	High	Fatty_liver	231	High	223	52	High
GS153	M	65	163.4	61.7	23.1089954	Normal	204.1	High	87.5	High	Fatty_liver	138	Low	177	52	High
GS019	F	61	150.8	57.6	25.3291016	High	213.1	High	91.5	High	Fatty_liver	287	High	162	53	High
GS059	M	58	164.7	68.1	25.1049824	High	212.7	High	88	High	Normal_liver	204	High	179	55	High
GS085	M	53	160.1	74.1	28.9091644	High	206.7	High	94	High	Fatty_liver	98	Low	182	56	High
GS067	M	44	172.7	76.6	25.6829014	High	227.5	High	92.5	High	Fatty_liver	105	Low	173	60	High
GS096	M	41	178	89.5	28.2476959	High	218.9	High	97	High	Fatty_liver	84	Low	197	60	High
GS126	M	52	170.4	78.4	27.0008164	High	256.9	High	97	High	Fatty_liver	200	High	336	60	High
GS064	M	43	173.4	78.7	26.1743889	High	227.4	High	98	High	Fatty_liver	194	High	202	68	High
GS143	M	44	164.8	68.9	25.3690853	High	223.9	High	87	High	Fatty_liver	341	High	210	75	High
GS102	M	51	171	85.7	29.3081627	High	255.9	High	106.5	High	Fatty_liver	157	High	228	76	High
GS035	M	48	175.4	85.5	27.7911758	High	203.4	High	95	High	Fatty_liver	447	High	206	83	High
GS008	M	44	166.4	80.4	29.0368443	High	215.7	High	92	High	Fatty_liver	184	High	220	89	High
GS141	F	50	151.4	82.8	36.1225662	High	191.2	High	103	High	Fatty_liver	180	High	153	89	High
GS108	F	38	161.5	56.7	21.7389221	Low	52.7	Low	77	Low	Normal_liver	55	Low	159	116	High
GS069	M	58	167.6	76.3	27.1629238	High	215.5	High	96.5	High	Normal_liver	69	Low	233	118	High
GS027	M	59	174.3	77.9	25.6414566	High	229.6	High	97.5	High	Fatty_liver	97	Low	226	130	High
GS002	M	54	179.9	86.2	26.6345234	High	256.7	High	100.5	High	Normal_liver	180	High	162	134	High
GS066	M	51	170.1	77.9	26.9233341	High	210.6	High	92	High	Normal_liver	111	Low	164	140	High
GS079	M	54	159.7	64.2	25.1724339	High	274.2	High	89.5	High	Normal_liver	194	High	233	168	High
GS160	M	46	167.5	60.2	21.4568939	Low	90.4	Low	78.5	Low	Normal_liver	164	High	318	307	High
