# Supplementary material for: Bifidobacterial carbohydrate/nucleoside metabolism enhances oxidative phosphorylation in white adipose tissue to protect against diet-induced obesity
Source: Microbiome. 2022 Nov 4;10:188. doi: 10.1186/s40168-022-01374-0 (PMC9635107; doi:10.1186/s40168-022-01374-0)
Supplement: Supplementary file 15 — Additional file 14: Supplementary Table 2. Primer list for qRT-PCR analysis. [file 40168_2022_1374_MOESM14_ESM.pdf]

Primer list for qRT-PCR analysis

	Primers for qRT-PCR analysis	
	Sequnce	
	Forward	Reverse
36B4	5′-CGTCCTCGTTGGAGTGACA-3′	5′-CGGTGCGTCAGGGATTG-3′
Acaca	5′-ATGGGCGGAATGGTCTCTTTC-3′	5′-TGGGGACCTTGTCTTCATCAT-3′
Acacb	5′-CGCTCACCAACAGTAAGGTGG-3′	5′-GCTTGGCAGGGAGTTCCTC-3′
Acadm	5′-AGGGTTTAGTTTTGAGTTGACGG-3′	5′-CCCCGCTTTTGTCATATTCCG-3′
Acc	5′-GGACAGACTGATCGCAGAGAAAG-3′	5′-TGGAGAGCCCCACACACA-3′
Aox	5′-TAACTTCCTCACTCGAAGCCA-3′	5′-AGTTCCATGACCCATCTCTGTC-3′
Chrebp	5′-CATTGCCAACATAAGCATCTTC-3′	5′-GTCCGATATCTCCGACACACTC-3′
Cidec	5′-ATGGACTACGCCATGAAGTCT-3′	5′-CGGTGCTAACACGACAGGG-3′
Cyp27a1	5′-CCAGGCACAGGAGAGTACG-3′	5′-GGGCAAGTGCAGCACATAG-3′
Cyp7a1	5′-TCATTGCTTCAGGGCTCCTG-3′	5′-TGGGCATCTCAAGCAAACAC-3′
Dgat2	5′-GCGCTACTTCCGAGACTACTT-3′	5′-GGGCCTTATGCCAGGAAACT-3′
Dio2	5′-AATTATGCCTCGGAGAAGACCG-3′	5′-GGCAGTTGCCTAGTGAAAGGT-3′
F4/80	5′-TGACAACCAGACGGCTTGTG-3′	5′-GCAGGCGAGGAAAAGATAGTGT-3′
Fasn	5′-GCTGCGGAAACTTCAGGAAAT-3′	5′-AGAGACGTGTCACTCCTGGACTT-3′
Fgf-15	5'-ACGTCCTTGATGGCAATCG-3'	5'-GAGGACCAAAACGAACGAAATT-3'
G0s2	5′-TAGTGAAGCTATACGTTCTGGGC-3′	5′-GTCTCAACTAGGCCGAGCA-3′
GcK	5′-CTGGATGACAGAGCCAGGATG-3′	5′-AGTTGGTTCCTCCCAGGTCT-3′
Ibabp	5′-CAAGGCTACCGTGAAGATGGA-3′	5′-CCCACGACCTCCGAAGTCT-3′
Ifn γ	5′-ATGAACGCTACACACTGCATC-3′	5′-CCATCCTTTTGCCAGTTCCTC-3′
Il-18	5′-CAGGCCTGACATCTTCTGCAA-3	5′-TCTGACATGGCAGCCATTGT-3′
Il-1 β	5′-CGGCACACCCACCCTG-3′	5′-AAACCGCTTTTCCATCTTCTTCT-3′
Mcp1	5′-GGCTCAGCCAGATGCAGTTAAC-3′	5′-AGCCTACTCATTGGGATCATCTTG-3′
Ost β	5'-GTATTTTCGTGCAGAAGATGCG-3'	5′-TTTCTGTTTGCCAGGATGCTC-3′
Pepck	5′-AAAAGCCTTTGGTCAACAAC-3′	5′-AAACTTCATCCAGGCAATGT-3′
Pgc1 α	5′-TATGGAGTGACATAGAGTGTGCT-3′	5′-CCACTTCAATCCACCCAGAAAG-3′
Plin2	5′-GACCTTGTGTCCTCCGCTTAT-3′	5′-CAACCGCAATTTGTGGCTC-3′
Ppar γ	5′-TCGCTGATGCACTGCCTATG-3′	5′-GAGAGGTCCACAGAGCTGATT-3′
Scd1	5′-TTCTTGCGATACACTCTGGTGC-3′	5′-CGGGATTGAATGTTCTTGTCGT-3′
Srebp1c	5′-GAAGCTGTCGGGGTAGCGTCT-3′	5′-CTCTCAGGAGAGTTGGCACCTG-3′
Tgf β	5′-CTTCAATACGTCAGACATTCGGG-3′	5′-GTAACGCCAGGAATTGTTGCTA-3′
Ucp1	5′-AGGCTTCCAGTACCATTAGGT-3′	5′-CTGAGTGAGGCAAAGCTGATTT-3′
